# Scoping review on socioemotional skills in the prevention of suicidal behavior among adolescents

**DOI:** 10.1590/0102-311XEN002524

**Published:** 2024-08-26

**Authors:** Joviana Quintes Avanci, Aline Ferreira Gonçalves, Orli Carvalho da Silva, Pedro Henrique Tavares, Simone Gonçalves de Assis

**Affiliations:** 1 Escola Nacional de Saúde Pública Sergio Arouca, Fundação Oswaldo Cruz, Rio de Janeiro, Brasil.; 2 Instituto Nacional de Saúde da Mulher, da Criança e do Adolescente Fernandes Figueira, Fundação Oswaldo Cruz, Rio de Janeiro, Brasil.

**Keywords:** Suicide, Self Injury, Suicidal Ideation, Adolescent, Suicide Prevention, Suicidio, Autolesiones, Ideación Suicida, Adolescente, Prevención del Suicidio

## Abstract

Promoting socioemotional skills has been highlighted among the evidence to prevent suicidal behavior in childhood and adolescence. This review aimed to map and analyze national and international scientific papers on initiatives and programs for the prevention of suicidal behavior in adolescence based on the theoretical framework of socioemotional skills. It is a scoping review using the methodology proposed by the Joanna Briggs Institute. Eleven academic bibliographic databases were analyzed, and searches were conducted on institutional websites related to suicide prevention and Google. Papers in Portuguese, Spanish, French, and English from 2010 to July 2022 were included in the review, which consisted of 97 studies, analyzed through data matrix and thematic grouping. The results show that most are international and focused on suicide, not on self-harm alone. In general, they have an informational and instructional bias for professionals, institutions, and governments, proposed laws, programs and action plans, studies on the role of socioemotional skills and intervention research. Few strategies have been clearly tested and validated. The key elements are the ability to perceive, recognize, understand, express, and regulate one’s own emotions, get motivated, and build empathy in relationships. Schools are key players in this process and the health system should act as a collaborative network. National and local prevention plans are required, emphasizing the role of schools, the health sector, and intersectoral coordination to promote health and quality of life.

## Resumen

El fomento de las habilidades socioemocionales se viene destacando entre las evidencias de prevención de la conducta suicida en la infancia y la adolescencia. Este artículo tiene como objetivo identificar y analizar la producción científica nacional e internacional sobre iniciativas y programas de prevención de la conducta suicida en la adolescencia a partir del marco teórico de las habilidades socioemocionales. Se trata de una revisión de alcance que utiliza la metodología propuesta por el Instituto Joanna Briggs. Se realizaron búsquedas en 11 bases de datos académicas, en sitios *web* institucionales relacionados con la prevención del suicidio y en Google. Los textos incluidos estaban publicados en portugués, español, francés o inglés, en el período entre 2010 y julio de 2022. La muestra consistió en 97 publicaciones, y se utilizaron la matriz de datos y agrupación temática para analizarlas. Los resultados muestran que la mayoría de las iniciativas son internacionales y dirigidas al suicidio, sin privilegiar la autolesión. En general, tienen un sesgo informativo e instruccional dirigido a profesionales, instituciones y gobiernos, proyecto de ley, programas y planes de acción, estudios sobre el papel de las habilidades socioemocionales e investigación de intervención. Pocas estrategias habían sido probadas y validadas claramente. Los elementos clave fueron la capacidad de percibir, reconocer, comprender, expresarse y regular las propias emociones, motivarse y establecer relaciones de empatía. Las escuelas son las protagonistas, y el sector salud necesita actuar en una red de colaboración. Se necesitan planes de prevención nacionales y locales, que pongan énfasis en el papel de la escuela, del sector salud y de la articulación intersectorial para la promoción de la salud y la calidad de vida.

## Introduction

Promoting socioemotional skills has been highlighted among the evidence to prevent suicidal behavior in childhood and adolescence, besides limited access to means of suicide, interaction with media channels for reporting, information, and care; and early identification and monitoring ^1^ ^,^ ^2^ ^,^ ^3^. Theories about suicide propose that adolescents who are unable to handle negative affect have their cognitive evaluation impacted, which leads to negative thoughts or expectations about the future and the perception of suicide as the only way out ^4^ ^,^ ^5^. In addition, the lack of social connections and a feeling of belonging and, instead, the presence of feelings of failure and imprisonment control the emotional state. This way, the lack of awareness and understanding of emotions, the inability to control impulsive behavior and use resolution strategies, as well as the existence of pain combined with hopelessness, can be conditions for the development of suicidal thoughts ^6^.

This way, many promising interventions with families, schools or in health services are based on socioemotional skills, prioritizing emotion regulation, problem resolution, and expansion of relational capacity ^1^ ^,^ ^7^. These skills include socioaffective, emotional, behavioral, and moral aspects. Among the theoretical constructs are intelligence and emotion regulation, which can be protective factors against emotional instability; difficult adaptation; guilt; feelings of failure, frustration, fear, and impulsiveness ^8^ ^,^ ^9^ ^,^ ^10^ ^,^ ^11^ ^,^ ^12^. They may receive interventions and can be taught, learned, and practiced over time, in order to achieve a sense of well-being and better social interaction. The “deficit” in social skills and in regulation of one’s own emotions seems to precede suicidal behaviors, where the search for the act can provide a false sense of relief ^13^ ^,^ ^14^.

Suicidal behavior refers to an action of self-harm or that could end one’s own life ^15^. It occurs in the form of suicidal ideation, planning or attempt, as well as self-harm, which may not be related to suicidal intent ^15^. In general, there is a fine line between these actions ^14^ ^,^ ^16^ ^,^ ^17^. Suicide rates in adolescence are alarming, representing the number four leading cause of death among young people aged 15 to 29 in the world ^18^. In Brazil, there has been an increasing trend in suicide since 2000 ^19^ ^,^ ^20^. Gender, age, and race/ethnicity have important implications for the epidemiology of suicide, especially when they show contexts of social vulnerability, discrimination, and violence ^21^. It is a multifactorial phenomenon, involving biological, social, psychological and philosophical-existential factors ^22^. Virtual social media have been highlighted in this debate for providing both risk and protection ^23^.

Considering the expansion of this problem in Brazil and other regions of the world, particularly among adolescents, the World Health Organization (WHO) and national bodies have been invited to develop prevention strategies to ensure access to public health services, strengthen policies and funding for vulnerable locations and populations, and offer comprehensive treatment with an emphasis on preventive actions ^13^ ^,^ ^14^ ^,^ ^16^ ^,^ ^24^ ^,^ ^25^ ^,^ ^26^ ^,^ ^27^ ^,^ ^28^ ^,^ ^29^. Given the relevance of socioemotional aspects, this study aims to map and analyze national and international scientific papers on initiatives and programs to prevent suicidal behavior in adolescence based on the theoretical framework of socioemotional skills in order to support actions that can be implemented in education, health, governmental and non-governmental services, and the media.

## Method

### Study design

This is a scoping review, a systematic method to map scientific papers on a given topic in order to identify concepts and gaps. The methodological framework was based on the Joanna Briggs Institute manual ^30^ and included papers with a variety of study methods and document sources. The following question guided this review: “How are strategies and programs on socioemotional skills developed to prevent suicidal behavior in childhood and adolescence?”. A review protocol was created, registered in the Open Science Framework (OSF) ^31^, and followed the PRISMA (*Preferred Reporting Items for Systematic Reviews and Meta-Analyses*) guidelines ^32^.

### Search strategies

Search was conducted between January and July 2022 and included two stages organized according to the type of source: (i) focused on academic databases, which included 11 bibliographic databases: SciELO, VHL Regional Portal, VHL Regional Portal (Health Science Descriptors - DeCS, acronym in Portuguese), OASIS (from Brazilian Institute of Information in Science and Technology - IBICT, acronym in Portuguese), Scopus, Web of Science, PubMed/MEDLINE (title/abstract), PubMed (MeSH terms), Dimensions, Embase (Emtree), PsycNET; and Google Scholar; and (ii) a search on institutional websites related to suicide prevention and Google.

Structured search was adapted for each database investigated and type of source by an experienced librarian using the following descriptors: “prevenção”, “suicídio” (“comportamento suicida” OR “ideação suicida” OR “suicídio” OR “autolesão” OR “comportamento autodestrutivo”), “inteligência emocional” (“manejo emocional” OR “regulação emocional” OR “controle emocional” OR “autocontrole emocional” OR “gerenciamento emocional”), “adolescente,” and “criança” (Box 1). Papers in Portuguese, Spanish, French, and English were included, with the time frame of 2010 to July 2022. Primary studies, secondary studies, experience reports, theoretical essays, theses, dissertations, official documents, reports, among others were part of this review.

**Box 1:** Description of bibliographic databases according to search terms and number of studies found, 2010-2022.

BIBLIOGRAPHICS BASES	SEARCH TERMS	PUBLICATIONS
SciELO: website that offers free access to journals. Integrated search for articles from journals in the SciELO network: Argentina, Brazil, Chile, Colombia, Cuba, Spain, Portugal, Venezuela, Public Health, Social Sciences. SciELO is an electronic library with a selected collection of scientific journals. SciELO is the result of a research project by São Paulo State Research Foundation (FAPESP, acronym in Portuguese), in partnership with the Latin American and Caribbean Center on Health Sciences Information (BIREME, acronym in Portuguese).	(“inteligência emocional” OR “manejo emocional” OR “regulação emocional” OR “controle emocional” OR “autocontrole emocional” OR “Gerenciamento emocional” OR “Emotional Intelligence” OR “Emotional Intelligences” OR “Intelligence, Emotional” OR “Intelligence, Social” OR “Intelligences, Emotional” OR “Intelligences, Social” OR “Social Intelligence” OR “Social Intelligences”) AND (“comportamento suicida” OR “ideação suicida” OR suicídio OR Suicidal OR “ehavior de suicídio” OR autolesão OR “Comportamento Autodestrutivo” OR “Self-Injurious Behavior” OR “Conducta Autodestructiva” OR “Deliberate Self Harm” OR “Deliberate Self-Harm” OR “Harm, Self” OR “Intentional Self Harm” OR “Intentional Self Injuries” OR “Intentional Self Injury” OR “Non Suicidal Self Injury” OR “Non-Suicidal Self Injuries” OR “Non-Suicidal Self Injury” OR “Nonsuicidal Self Injuries” OR “Nonsuicidal Self Injury” OR “Self Destructive Behavior” OR “Self Harm” OR “Self Harm, Intentional” OR “Self Injuries, Non-Suicidal” OR “Self Injuries, Nonsuicidal” OR “Self Injurious Behavior” OR “Self Injury” OR “Self Injury, Intentional” OR “Self Injury, Non-Suicidal” OR “Self Injury, Nonsuicidal” OR “Self-Destructive Behavior” OR “Self-Destructive Behaviors” OR “Self-Harm, Deliberate” OR “Self-Injuries” OR “Self-Injurious Behaviors” OR “Self-Injury” OR “Autoagressão Intencional” OR “Conduta Autolesiva” OR “Ferimento Autoinfligido não Suicida” OR “Lesão Autoinfligida não Suicida”)) AND (Adolesc* OR adolescência OR Teenager) AND (Prevenção OR Prevention OR Prevención)	0
VHL Regional Portal (title, abstract, and subject): Integrated search in BIREME databases: LILACS - Latin American and Caribbean Health Sciences Literature MEDLINE - International Health Sciences Literature ADOLEC - Adolescent Health ADSAUDE - Health Services Administration BBO - Brazilian Bibliography of Dentistry BDENF - Nursing Database BIOÉTICA - Pan American Health Organization (PAHO)/World Health Organization (WHO) Regional Bioethics Program Database DESASTRES - Disaster Documentation Center Collection HISA - History of Public Health in Latin America and the Caribbean HOMEOINDEX - Brazilian Bibliography of Homeopathy LEYES - Basic Health Legislation of Latin America and the Caribbean MEDCARIB - Caribbean Literature in Health Sciences REPIDISCA - Literature in Sanitary Engineering and Environmental Sciences PAHO - Collection of the Library of the PAHO WHOLIS - WHO Library Information System	(“inteligência emocional” OR “manejo emocional” OR “regulação emocional” OR “controle emocional” OR “autocontrole emocional” OR “Gerenciamento emocional” OR “Emotional Intelligence” OR “Emotional Intelligences” OR “Intelligence, Emotional” OR “Intelligence, Social” OR “Intelligences, Emotional” OR “Intelligences, Social” OR “Social Intelligence” OR “Social Intelligences”) AND (“comportamento suicida” OR “ideação suicida” OR suicídio OR suicidal OR “ehavior de suicídio” OR autolesão OR “Comportamento Autodestrutivo” OR “Self-Injurious Behavior” OR “Conducta Autodestructiva” OR “Deliberate Self Harm” OR “Deliberate Self-Harm” OR “Harm, Self” OR “Intentional Self Harm” OR “Intentional Self Injuries” OR “Intentional Self Injury” OR “Non Suicidal Self Injury” OR “Non-Suicidal Self Injuries” OR “Non-Suicidal Self Injury” OR “Nonsuicidal Self Injuries” OR “Nonsuicidal Self Injury” OR “Self Destructive Behavior” OR “Self Harm” OR “Self Harm, Intentional” OR “Self Injuries, Non-Suicidal” OR “Self Injuries, Nonsuicidal” OR “Self Injurious Behavior” OR “Self Injury” OR “Self Injury, Intentional” OR “Self Injury, Non-Suicidal” OR “Self Injury, Nonsuicidal” OR “Self-Destructive Behavior” OR “Self-Destructive Behaviors” OR “Self-Harm, Deliberate” OR “Self-Injuries” OR “Self-Injurious Behaviors” OR “Self-Injury” OR “Autoagressão Intencional” OR “Conduta Autolesiva” OR “Ferimento Autoinfligido não Suicida” OR “Lesão Autoinfligida não Suicida”) AND (adolesc* OR adolescência OR teenager OR Criança* OR Niños OR Child OR Chiildhood OR Infancy) AND (prevenção OR prevention OR prevención) AND (year_cluster:[2010 TO 2022])	30
VHL Regional Portal (Health Science Descriptors) (same as above)	(e☹ “Inteligência Emocional” OR )) AND (e☹Suicídio OR “Comportamento Autodestrutivo” OR “Tentativa de suicídio”)) AND (e☹ Adolescen* OR Crianças)) AND (e☹ Prevenção OR Preventivo))	0
OASIS (from Brazilian Institute of Information in Science and Technology - IBICT, acronym in Portuguese): Brazilian portal for open access repositories and journals. Through a single interface, it allows simultaneous search in all digital repositories and electronic scientific journals that use the OAI-PMH protocol, which makes it a service provider. In other words, data providers (institutions, scientific journals) expose the metadata that describe their content so that they can be collected by the service provider, which centralizes search services. IBICT developed and coordinates the Brazilian Digital Library of Theses and Dissertations (BDTD, acronym in Portuguese), which integrates the information systems for theses and dissertations in educational and research institutions in Brazil; it encourages the electronic registration and publication of theses and dissertations. The BDTD, in partnership with Brazilian educational and research institutions, enables the Brazilian science and technology community to publish and disseminate its theses and dissertations produced in the country and abroad, increasing the visibility of national scientific production.	(“inteligência emocional” OR “manejo emocional” OR “regulação emocional” OR “controle emocional” OR “autocontrole emocional” OR “Gerenciamento emocional” OR “Emotional Intelligence” OR “Emotional Intelligences” OR “Intelligence, Emotional” OR “Intelligence, Social” OR “Intelligences, Emotional” OR “Intelligences, Social” OR “Social Intelligence” OR “Social Intelligences”) AND (“comportamento suicida” OR “ideação suicida” OR suicídio OR Suicidal OR “ehavior de suicídio” OR autolesão OR “Comportamento Autodestrutivo” OR “Self-Injurious Behavior” OR “Conducta Autodestructiva” OR “Deliberate Self Harm” OR “Deliberate Self-Harm” OR “Harm, Self” OR “Intentional Self Harm” OR “Intentional Self Injuries” OR “Intentional Self Injury” OR “Non Suicidal Self Injury” OR “Non-Suicidal Self Injuries” OR “Non-Suicidal Self Injury” OR “Nonsuicidal Self Injuries” OR “Nonsuicidal Self Injury” OR “Self Destructive Behavior” OR “Self Harm” OR “Self Harm, Intentional” OR “Self Injuries, Non-Suicidal” OR “Self Injuries, Nonsuicidal” OR “Self Injurious Behavior” OR “Self Injury” OR “Self Injury, Intentional” OR “Self Injury, Non-Suicidal” OR “Self Injury, Nonsuicidal” OR “Self-Destructive Behavior” OR “Self-Destructive Behaviors” OR “Self-Harm, Deliberate” OR “Self-Injuries” OR “Self-Injurious Behaviors” OR “Self-Injury” OR “Autoagressão Intencional” OR “Conduta Autolesiva” OR “Ferimento Autoinfligido não Suicida” OR “Lesão Autoinfligida não Suicida”) AND (Adolesc* OR adolescência OR Teenager OR Criança* OR Niños OR Child OR Chiildhood OR Infancy) AND (Prevenção OR Prevention OR Prevención)	4
Scopus: references with abstracts. Scopus is a comprehensive scientific, medical, technical and social science database containing all relevant literature.	(TITLE-ABS-KEY (“Emotional Intelligence” OR “Emotional Intelligences” OR “Intelligence, Emotional” OR “Intelligence, Social” OR “Intelligences, Emotional” OR “Intelligences, Social” OR “Social Intelligence” OR “Social Intelligences” ) AND TITLE-ABS-KEY (suicidal OR “Suicide ideation” OR “Self-Injurious Behavior” OR “Behavior, Self-Destructive” OR “Behavior, Self-Injurious” OR “Behaviors, Self-Destructive” OR “Behaviors, Self-Injurious” OR “Deliberate Self Harm” OR “Deliberate Self-Harm” OR “Harm, Self” OR “Intentional Self Harm” OR “Intentional Self Injuries” OR “Intentional Self Injury” OR “Non Suicidal Self Injury” OR “Non-Suicidal Self Injuries” OR “Non-Suicidal Self Injury” OR “Nonsuicidal Self Injuries” OR “Nonsuicidal Self Injury” OR “Self Destructive Behavior” OR “Self Harm” OR “Self Harm, Intentional” OR “Self Injuries, Non-Suicidal” OR “Self Injuries, Nonsuicidal” OR “Self Injurious Behavior” OR “Self Injury” OR “Self Injury, Intentional” OR “Self Injury, Non-Suicidal” OR “Self Injury, Nonsuicidal” OR “Self-Destructive Behavior” OR “Self-Destructive Behaviors” OR “Self-Harm, Deliberate” OR “Self-Injuries” OR “Self-Injurious Behaviors” OR “Self-Injury”) AND TITLE-ABS-KEY (adolesc* OR teenager OR child OR infancy OR kids OR children) AND TITLE-ABS-KEY (prevention))	12
Web of Science: a multidisciplinary database that indexes the most cited journals in their respective fields. It is also a citation index, providing, for each article, information about cited documents and documents that have cited the article. Today, it has more than 9,000 journals indexed. It comprises: Science Citation Index Expanded (SCI-EXPANDED): 1945 to the present; Social Sciences Citation Index: 1956 to the present; Arts and Humanities Citation Index: 1975 to the present. In 2012, the content was expanded with the inclusion of the Conference Proceedings Citation Index ₋ Science (CPCI-S); Conference Proceedings Citation Index ₋ Social Science & Humanities (CPCI-SSH)	“Emotional Intelligence” OR “Emotional Intelligences” OR “Intelligence, Emotional” OR “Intelligence, Social” OR “Intelligences, Emotional” OR “Intelligences, Social” OR “Social Intelligence” OR “Social Intelligences” (Todos os campos) and Suicidal OR “Suicide ideation” OR “Self-Injurious Behavior” OR “Behavior, Self-Destructive” OR “Behavior, Self-Injurious” OR “Behaviors, Self-Destructive” OR “Behaviors, Self-Injurious” OR “Deliberate Self Harm” OR “Deliberate Self-Harm” OR “Harm, Self” OR “Intentional Self Harm” OR “Intentional Self Injuries” OR “Intentional Self Injury” OR “Non Suicidal Self Injury” OR “Non-Suicidal Self Injuries” OR “Non-Suicidal Self Injury” OR “Nonsuicidal Self Injuries” OR “Nonsuicidal Self Injury” OR “Self Destructive Behavior” OR “Self Harm” OR “Self Harm, Intentional” OR “Self Injuries, Non-Suicidal” OR “Self Injuries, Nonsuicidal” OR “Self Injurious Behavior” OR “Self Injury” OR “Self Injury, Intentional” OR “Self Injury, Non-Suicidal” OR “Self Injury, Nonsuicidal” OR “Self-Destructive Behavior” OR “Self-Destructive Behaviors” OR “Self-Harm, Deliberate” OR “Self-Injuries” OR “Self-Injurious Behaviors” OR “Self-Injury” (Todos os campos) and Adolesc* OR Teenager OR child OR infancy OR kids OR children (Todos os campos) and Prevention (Todos os campos).	11
PubMed/MEDLINE (title and abstract): database specializing in biomedical and life sciences developed by the U.S. National Institutes of Health (NIH) and managed by the National Center for Biotechnology Information (NCBI). Of public access, it indexes specialized literature in the fields of biological sciences, nursing, dentistry, medicine, veterinary medicine, and public health	(((((“emotional intelligence”[Title/Abstract]) OR (“social intelligence”[Title/Abstract])) OR (Intelligence,[Title/Abstract])) AND (((“suicidal behavior”[Title/Abstract]) OR (“suicide”[Title/Abstract])) OR (“self destructive behavior”[Title/Abstract]))) AND ((((“adolescence”[Title/Abstract]) OR (“adolescent”[Title/Abstract])) OR (“child”[Title/Abstract])) OR (“kids”[Title/Abstract]))) AND (“prevention”[Title/Abstract])	8
PubMed (MeSH terms): same as above	(((“emotional intelligence”[MeSH Terms] AND (2010/1/1:2022/5/28[pdat])) AND ((“self injurious behavior”[MeSH Terms]) OR (“suicide”[MeSH Terms]) AND (2010/1/1:2022/5/28[pdat]))) AND ((“adolescent”[MeSH Terms]) OR (“child”[MeSH Terms]) AND (2010/1/1:2022/5/28[pdat]))) AND (prevention AND (2010/1/1:2022/5/28[pdat]))	96
Dimensions: this is a database that offers comprehensive collections of linked data on a single platform, from funding, publications, datasets, and clinical trials to patents and policy documents. It maps the life cycle of research, from funding to results and impacts	(“Emotional Intelligence” OR “Social intelligence”) AND (“Self destructive behavion” OR “Self injuries” OR “Self injurious behavior”) OR (Suicide OR Suicidal) OR (Adolescent Or Adolescence OR Adolescents OR Child OR Childhood OR Kids OR Children OR infancy) AND (Prevention OR Preventive).	24
Embase (Emtree): it is considered a reference database in the field of biomedical and pharmacological responses. Its website presents the benefits this tool in evidence-based medicine, contributing to evidence-based clinical decision-making, improving patient outcomes, increasing the discovery of biomedical evidence, and providing comprehensive updated biomedical information; in pharmacovigilance, contributing to the literature in this field; in medical device development and post-market surveillance, contributing to the stages of medical device development with high-quality biomedical information, from concept and design to post-market surveillance; in drug development, contributing to the discovery of relationships between drugs, diseases, and drug interactions, providing critical biomedical information for drug development, repositioning and safety. This database provides systematic and integrative review studies, clinical guidelines and protocols, and health technology assessment. The entire process of systematic and integrative reviews has internationally recognized guidelines. It also includes more than 2.3 million abstracts of conference papers since 2009. With daily updates and annual inclusion of more than 1.5 million articles, it offers PICO Search, a unique feature that allows searches based on the PICO (Patient, Intervention, Comparison and Outcome) strategy, a methodology used in evidence-based practice (EBP)	‘emotional intelligence’/exp AND (‘suicide’/exp OR ‘self destructive behavior’/exp OR ‘automutilation’/exp OR ‘suicidal behavior’/exp OR ‘suicide attempt’/exp) AND (‘adolescence’/exp OR ‘childhood’/exp OR ‘child’/exp OR ‘adolescent’/exp) AND prevention AND [2010-2022]/py	12
PsycNET: database for psychology, education, psychiatry, social sciences. PsycINFO is the world’s leading psychology database, covering virtually all literature available on these subjects. Updated weekly, it offers millions of abstracts of journal articles, book chapters, editorials, and other types of references and bibliographic citations provided by the most respected academic publications. It offers more than 4 million bibliographic citations (article abstracts). It currently has around 2,500 titles, 80% of the content from journals (99% of the journals available on PsycINFO are peer-reviewed). Comprehensible coverage from 1880 to the present, although it contains records dating back to 1597	“Emotional intelligence” OR Abstract: “social intelligence” AND Abstract: “Self injuries” OR Abstract: “Self Injurious behavior” OR Abstract: automultilation OR Abstract: Suicide OR Abstract: Suicidal AND Abstract: Adolesc* OR Abstract: Child* AND Abstract: Prevention OR Abstract: Preventive AND Year: 2010 To 2022	5
TOTAL		202
TOTAL WITHOUT REPEATED STUDIES		150

Google search was performed on pages from countries with high suicide mortality rates (≥ 2 per 100,000 inhabitants) in the 0-19 age group, according to the WHO (2021) (Table 1). The total number of pages was counted according to the country and organized by world region. The first 20 most relevant results were analyzed using the website found and/or documents related to the main website. In addition, institutional websites were investigated according to the recommendations of experts and information on the websites initially found ^33^. A simplified version of search terms was used here: “*inteligência emocional*” (emotional intelligence), “*regulação emocional*” (emotion regulation), “*habilidade socioemocional*” (socioemotional skill), “*suicídio*” (suicide), and “*autolesão*” (self-harm).

**Table 1:** Regions with the highest suicide mortality rates in childhood and adolescence and availability of information on emotional intelligence/related skills and suicide/self-harm on Google pages of each country.

Region	Countries and regions (WHO)	Countries with high rates * n (%)	Results found on the 1st page	Exclusions	Included in final spreadsheet
Africa	47	27 (57.5)	16	16	0
Americas	33	21 (63.6)	93	78	15
Europe	50	39 (78.0)	101	97	4
East Mediterranean	21	8 (38.1)	5	5	0
Western Pacific	21	11 (52.4)	41	35	6
Southwest Asia	11	8 (72.7)	19	19	0
Total	183	114 (62.3)	275	250	25

WHO: World Health Organization.

* Countries with high rates per 100,000 inhabitants are those with rates ≥ 2/100,000 in the 5-14 age group and/or > 5.5/100,000 in the 15-29 age group in 2012.

### Selection of studies and eligibility criteria

The initial search presented the following numbers of papers: (i) 202 from academic databases, (ii) 300 documents on Google Scholar, (iii) 275 on the Google pages of countries with high suicide rates among children and adolescents, and (iv) 44 on institutional websites.

After excluding duplicates, selection was made in pairs using Rayyan (https://www.rayyan.ai/) ^34^ reference manager for the initial screening of abstracts and titles. The evaluators worked independently and the individual results were checked by a third expert. Disagreements were resolved by consensus. The following exclusion criteria were applied: absence of a relationship between socioemotional skills and suicidal/self-harm behavior in childhood or adolescence; an age group other than childhood or adolescence; specific approach to the topics of interest; documents and websites not available; works without access to full text; commercial websites that sell books or texts; absence of the text in Portuguese, English, French or Spanish; and audiovisual products such as videos and podcasts. Eligible studies were those that addressed the themes of socioemotional skills in relation to suicidal/self-harm behavior, with a focus on prevention in childhood and adolescence.

**Figure 1:**
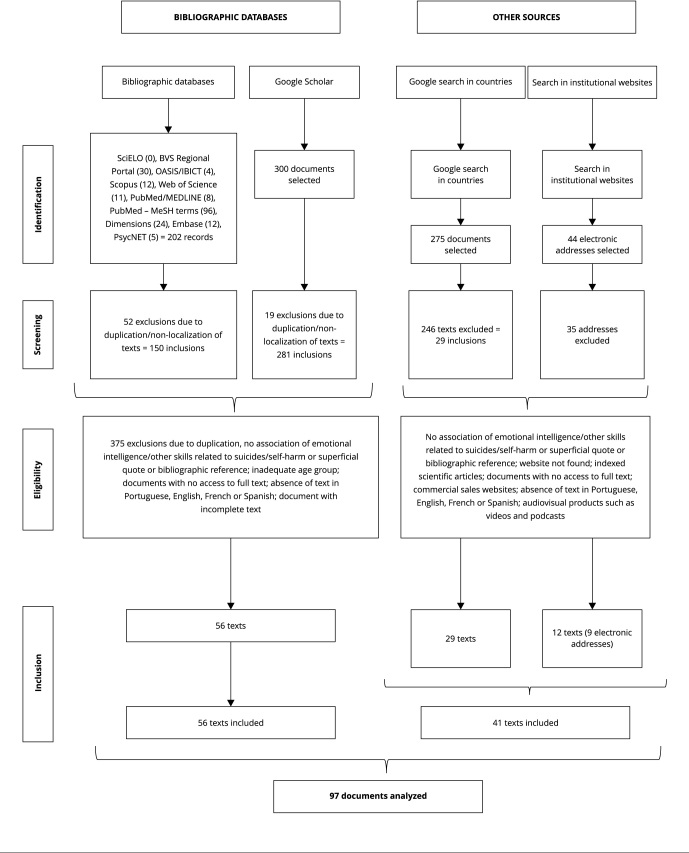
Steps of identification, screening, eligibility and inclusion of documents in the review according to data collection sources.

Figure 1 shows the stages of identification and selection of papers, which totaled 97 papers. Of these, 56 were from bibliographic databases and 41 resulted from a combination of 29 documents from Google search and 12 from search on websites of institutions. Most documents from non-bibliographic databases are government guides, protocols or reports published for scientific dissemination, followed by texts from websites, end-of-course papers, books, book chapters or extended texts presented at congresses, online reports, and one proposed law.

### Analysis of studies

All 97 studies were analyzed using two analytical methods: (i) characterization of papers by means of a data matrix describing the studies according to title, author, abstract, country, year of publication, target audience, main concepts, objectives, and methods applied; and (ii) thematic grouping according to actions/strategies/programs for schools, health services, governmental and nongovernmental services, and media.

## Results

### Characterization of papers

Box 2 shows that studies produced in the United States represent most papers analyzed (16.5%), followed by Brazil and Spain (12.3% each) ^3^ ^,^ ^12^ ^,^ ^35^ ^,^ ^36^ ^,^ ^37^ ^,^ ^38^ ^,^ ^39^ ^,^ ^40^ ^,^ ^41^ ^,^ ^42^ ^,^ ^43^ ^,^ ^44^ ^,^ ^45^ ^,^ ^46^ ^,^ ^47^ ^,^ ^48^ ^,^ ^49^ ^,^ ^50^ ^,^ ^51^ ^,^ ^52^ ^,^ ^53^ ^,^ ^54^ ^,^ ^55^ ^,^ ^56^ ^,^ ^57^ ^,^ ^58^ ^,^ ^59^ ^,^ ^60^ ^,^ ^61^ ^,^ ^62^ ^,^ ^63^ ^,^ ^64^ ^,^ ^65^ ^,^ ^66^ ^,^ ^67^ ^,^ ^68^ ^,^ ^69^ ^,^ ^70^, and in descending order: Colombia, Australia, Peru, Argentina, Switzerland, India, Mexico, Canada, Portugal, Costa Rica, the United Kingdom, Sweden, New Zealand, the Netherlands, Finland, Germany, Poland, Bolivia, Ecuador, Kenya, Malaysia, South Africa, and lastly, Egypt, China, and Turkey. Regarding the year of publication, a gradual increase has been observed in the number of studies since 2012, reaching the peak production in 2018 and maintaining an average of 15 studies per year between 2018 and 2021.

**Box 2:** Categorization of studies according to author, year of text publication/retrieval, country, socioemotional skills, and type of suicidal/self-harm behavior (n = 97).

STUDY	YEAR	COUNTRY	SOCIOEMOCIONALS SKILLS	SUICIDAL/SELF-HARM BEHAVIOR
Buerger et al. ^13^	2022	Germany	Emotion regulation	Self-harm
Garmendia Espinoza ^70^	2022	Spain	Emotional intelligence	Suicidal ideation
Schwartz ^122^	2022	Canada	Emotion regulation	Suicide
Nova Escola ^50^	2022	Brazil	Emotional intelligence	Suicide
Hermosillo-de-la-Torre et al. ^84^	2021	Mexico	Emotion regulation, self-esteem	Suicidal behavior
Knight ^44^	2021	United States	Emotional competences, positive attitudes, resilience etc.	Suicide
Shahram et al. ^114^	2021	Canada	Resilience, *coping*	Suicide
Massagli et al. ^55^	2021	Brazil	Socioemotionals skills	Violence
Rastrollo Sasal ^69^	2021	Spain	Emotion regulation, emotional skills	Suicidal behavior
Krishnamoorthy & Kalpana ^110^	2021	India	Emotional intelligence, emotional skills and competencies	Suicidal behavior
Cano Quevedo ^85^	2021	Peru	Emotional health	Self-harm
Arguedas González et al. ^123^	2021	Costa Rica	Emotional intelligence, social skills, self-esteem	Suicide, attempts, ideation
World Health Organization ^18^	2021	Switzerland	Socioemotionals skills	Suicide, self-harm
World Health Organization & United Nations Children’s Fund ^99^	2021	Switzerland	Emotional competencies, resilience	Suicide, self-harm
Scavacini et al. ^48^	2021	Brazil	Emotion regulation	Self-harm
Farias ^117^	2021	Argentina	Emotion regulation	Self-harm, suicidal ideation
Argentine Ministry of Health et al. ^102^	2021	Argentina	Emotional intelligence, emotion regulation, empathy etc.	Self-harm, suicide
Misiones Ministry of Public Health ^111^	2021	Argentina	Emotional intelligence	Suicide
SOMOS Educação ^53^	2021	Brazil	Emotional intelligence socioemotionals skills, resilience etc.	Suicide
Rodríguez ^112^	2021	Colombia	Emotional intelligence, self-esteem, empathy	Suicidal behavior
Colombian Senate ^104^	2021	Colombia	Emotional intelligence, empathy and emotion regulation	Suicide
Costarican National Council for Youth Public Policies ^96^	2021	Costa Rica	Resilience	Suicide
Kim et al. ^14^	2020	United States	Emotion regulation	Suicide attempt, self-harm
Acuña de la Cruz & Gamarra Zelada ^82^	2020	Peru	Emotional intelligence	Suicidal ideation
Bonet et al. ^63^	2020	Spain	Emotional intelligence, emotion regulation	Suicide
Aquino Huanca ^83^	2020	Bolivia	Emotional intelligence	Self-harm
Fernandez Moratilla ^64^	2020	Spain	Emotion regulation, resilience, etc.	Suicide
Arrivillaga et al. ^65^	2020	Spain	Emotional intelligence	Suicidal ideation
Pathare et al. ^90^	2020	India	Emotional intelligence	Suicide
Quintana-Orts et al. ^61^	2020	Spain	Emotion regulation, emotional intelligence	Suicidal ideation
Halicka et al. ^109^	2020	United States	Emotional intelligence	Self-harm, suicidal behavior
Velis Giménez ^67^	2020	Spain	Emotional intelligence, emotion regulation	Self-harm
Fonseca-Pedrero et al. ^68^	2020	Spain	Emotion regulation, self-esteem, skills	Suicidal behavior
Magalhães & Carrasco ^56^	2020	Brazil	Social skills	Suicidal behavior, self-harm
World Health Organization ^106^	2020	Switzerland	Resilience, emotional skills, emotion regulation etc.	Suicidal behavior, self-harm
Trew et al. ^100^	2020	Australia	Emotional intelligence, emotion regulation	Ideation, suicidal behavior
Australian Government. National Suicide Prevention Taskforce ^95^	2020	Australia	Emotional intelligence, emotion regulation, emotional well-being	Suicide
Brausch & Woods ^43^	2019	United States	Emotion regulation	Suicidal ideation, self-harm
Flores-Kanter et al. ^91^	2019	Spain	Emotional intelligence, emotion regulation	Suicidal ideation
Galarreta Mostacero ^81^	2019	Peru	Emotion regulation, social skills	Self-harm
Quintana-Orts et al. ^66^	2019	Spain	Emotional intelligence	Suicide
Rey et al. ^62^	2019	Spain	Emotional intelligence	Suicide
Bezerra ^57^	2019	Brazil	Emotional intelligence, emotional coefficient	Suicide
Vollandt ^87^	2019	United States	Socialemotional learning	Suicide
Scavacini et al. ^47^	2019	Brazil	Emotion regulation	Suicide, self-harm
Goodman et al. ^124^	2019	NI	Emotion regulation	Self-harm
National Suicide Prevention Taskforce ^94^	2019	Australia	Emotional intelligence	Suicide
Almeida & Almeida ^54^	2019	Brazil	Emotional intelligence, self-esteem, positive humor	Suicide
Colombian Ministry of Communication Technology and Information ^103^	2019	Colombia	Emotional intelligence	Suicide
Northern Territory Department of Health ^97^	2018/2023	Australia	Emotional intelligence	Suicide
Roberts ^41^	2018	United States	Resilience, self-rgulation etc.	Suicidal ideation
Ganaprakasam ^77^	2018	Malaysia	Emotional intelligence	Suicidal ideation
Cruz Cob et al. ^116^	2017	Mexico	Self-esteem etc.	Suicidal behavior
Gallagher & Miller ^42^	2018	United States	Emotional intelligence, self-esteem, emotion regulation etc.	Ideation and suicidal behavior
Sánchez ^59^	2018	Spain	Emotion regulation	Self-harm
Colorado ^118^	2018	Colombia	Attachment, emotion regulation	Suicidal behavior
Okello & Aomo ^78^	2018	Kenya	Emotional intelligence	Suicidal behavior
Zachariah et al. ^79^	2018	India	Emotional intelligence and behavior, emotion regulation	Suicide
Domínguez-García & Fernández-Berrocal ^60^	2018	Spain	Emotional intelligence	Suicidal behavior
Fernández ^80^	2018	Ecuador	Emotional intelligence	Self-destructive behavior
World Health Organization ^92^	2018	Switzerland	Emotion regulation	Suicide
Bloomer ^101^	2018	United Kingdom	Emotional intelligence	Self-harm
Brazilian Neuropsychology Society ^49^	2018	Brazil	Emotion regulation, empathy, resilience	Suicide
Sucena ^51^	2018	Brazil	Emotional intelligence, emotional well-being	Suicide
Senac Goiás ^52^	2018	Brazil	Emotional intelligence, socioemotionals skills, emotion regulation	Suicide
World Health Organization ^58^	2018	Switzerland	Life skills, resilience, emotion regulation	Self-harm, suicide
Towers Hamlets ^105^	2017/2018	United Kingdom	Emotional intelligence	Suicide
Alvino Advíncula & Huaytalla Pariona ^74^	2017	Peru	Emotional intelligence	Self-harm
Mohamed et al. ^89^	2017	Egypt	Emotional intelligence	Suicidal ideation
Du Plooy ^75^	2017	South Africa	Emotional intelligence	Suicidal behavior
Xavier ^76^	2017	Portugal	Emotional intelligence, emotion regulation	Self-harm
Topper ^40^	2017	United States	Emotional intelligence, resilience etc.	Suicidal behavior
Stone et al. ^3^	2017	United States	Emotion regulation	Suicide
Stern & Divecha ^93^	2017	Finland	Emotional intelligence	Suicide
González Suárez et al. ^113^	2016	Colombia	Emotional intelligence	Self-harm
Perloe ^38^	2016	United States	Emotional intelligence	Self-harm
Kaufman et al. ^45^	2016	United States	Emotion regulation	Suicide, self-harm
Black Dog Institute ^98^	2016	Australia	Emotional intelligence	Suicide
Benito et al. ^108^	2016	Argentina	Emotional intelligence, emotion regulation, empathy etc.	Self-harm, suicide
Romo et al. ^125^	2016	Sweden	Emotion management	Suicidal ideation
Valois et al. ^37^	2015	United States	Self-efficacy, emotional reactions	Suicidal ideation and suicide attempts
Kwok et al. ^73^	2015	China	Emotional intelligence and competencies, social problem solving	Suicidal ideation
Bodzy et al. ^39^	2015	United States	Emotional intelligence, emotion management	Suicidal ideation and suicide attempts
Wasserman et al. ^86^	2015	Sweden	Socioemotionals skills	Suicidal behavior
Purebl et al. ^2^	2015	The Netherlands	Emotional intelligence, emotion regulation	Suicide
Fuller et al. ^46^	2015	United States	Emotion regulation, social skills, self-esteem	Suicidal behavior, self-harm
Voon et al. ^71^	2014	Australia	Emotion regulation	Self-harm
Oktan ^72^	2014	Turkey	Self-care, autonomy, etc.	Self-harm
Santos et al. ^88^	2014	Portugal	Social skills, self-concept etc.	Suicidal behavior
Jacobson et al. ^36^	2013	United States	Emotion regulation, social communication	Suicide attempt
Appelhoff ^115^	2013	New Zealand	Emotional intelligence, emotional well-being, resilience	Suicide
Awasthi ^126^	2012	India	Emotional intelligence	Suicide
Suárez-Colorado ^127^	2012	Colombia	Emotional intelligence	Suicidal ideation, suicide attempt, suicide
Rolston & Lloyd-Richardson ^128^	ND	United States	Emotion regulation, *coping*	Self-harm
Department of Health and Human Services ^35^	ND	United States	Socialemotional learning	Suicide
Wasserman et al. ^129^	2012	NI	Emotion management	Suicidal behavior
Community-Led Action for Resilience ^107^	ND	Canada	Resilience	Suicide

ND: no date: NI: no information.

Most studies analyzed (32%) have actors from school institution as their target audience ^13^ ^,^ ^36^ ^,^ ^37^ ^,^ ^44^ ^,^ ^55^ ^,^ ^57^ ^,^ ^61^ ^,^ ^62^ ^,^ ^64^ ^,^ ^65^ ^,^ ^66^ ^,^ ^67^ ^,^ ^68^ ^,^ ^69^ ^,^ ^71^ ^,^ ^72^ ^,^ ^73^ ^,^ ^74^ ^,^ ^75^ ^,^ ^76^ ^,^ ^77^ ^,^ ^78^ ^,^ ^79^ ^,^ ^80^ ^,^ ^81^ ^,^ ^82^ ^,^ ^83^ ^,^ ^84^ ^,^ ^85^ ^,^ ^86^ ^,^ ^87^ ^,^ ^88^. Other studies address the theme in a clinical context ^14^ ^,^ ^38^ ^,^ ^39^ ^,^ ^89^, three show a community perspective ^41^ ^,^ ^90^ ^,^ ^91^ and only one has an institutional view, conducted with children under guardianship in Catalonia (Spain) ^63^. Also, most texts focus on adolescents, while some include children and young adults ^39^ ^,^ ^92^ ^,^ ^93^ ^,^ ^94^. The approach to the categories of gender, sexual orientation, race/ethnicity, and social class is restricted to a brief theoretical reflection, considered in sample constitution and in the descriptive analysis of the results. However, there is a consensus that minorities should be studied because they suffer more discrimination, isolation, exclusion or find obstacles to access any kind of support ^95^. Particular attention should be dedicated to the LGBTQIA+ population; black young people; Indigenous, rural, immigrant and refugee communities; adolescents deprived of their liberty, people with disabilities and those in foster care institutions ^2^ ^,^ ^40^ ^,^ ^90^ ^,^ ^94^ ^,^ ^95^ ^,^ ^96^ ^,^ ^97^ ^,^ ^98^ ^,^ ^99^ ^,^ ^100^ ^,^ ^101^.

Regarding the main concepts of socioemotional skills, a common theoretical basis was identified among the studies rooted in social emotional learning, emotion regulation, emotional intelligence, social and emotional intelligence, resilience, empathy, emotional skills, self-knowledge, emotional competence, promotion of self-esteem and mental health (Box 2). Of these, emotional intelligence and emotion regulation are highlighted. The key elements are the ability to perceive, recognize, understand, express, and regulate one’s own emotions, get motivated, recognize the emotions of other people, and build empathy in relationships. The expression of emotions is highly valued.

In general, the studiees can be organized as follows: (i) studies with an informational and instructional bias for professionals, institutions, and governments, addressing not only the prevention of suicidal/self-harm behavior, but also risk habits, mental disorders, promotion of mental well-being, care, coping with violence, and promotion of social emotional learning ^47^ ^,^ ^48^ ^,^ ^49^ ^,^ ^94^ ^,^ ^102^ ^,^ ^103^; (ii) proposed law, which takes emotional education as a framework integrated into the educational training process ^104^; (iii) programs and action plans ^2^ ^,^ ^3^ ^,^ ^46^ ^,^ ^58^ ^,^ ^95^ ^,^ ^96^ ^,^ ^97^ ^,^ ^98^ ^,^ ^99^ ^,^ ^101^ ^,^ ^102^ ^,^ ^105^ ^,^ ^106^ ^,^ ^107^; (iv) association studies aiming to understand the role of socioemotional skills in the development of suicidal behavior, whether as a risk or protection ^37^ ^,^ ^38^ ^,^ ^39^ ^,^ ^69^ ^,^ ^73^ ^,^ ^108^ ^,^ ^109^; and (v) intervention studies that seek to determine the effectiveness and applicability of interventions based on socioemotional skills and promotion of mental health in order to reduce suicidal/self-harm behavior ^13^ ^,^ ^14^ ^,^ ^57^ ^,^ ^60^ ^,^ ^70^ ^,^ ^86^ ^,^ ^88^ ^,^ ^90^ ^,^ ^110^.

Most studies are theoretical (55.6%), with an emphasis on suicide and self-harm prevention (Box 2). Risk behaviors, mental disorders, promotion of mental well-being, care, coping with violence against children and adolescents, and social emotional learning are also mentioned ^2^ ^,^ ^3^ ^,^ ^46^ ^,^ ^47^ ^,^ ^48^ ^,^ ^49^ ^,^ ^58^ ^,^ ^94^ ^,^ ^95^ ^,^ ^96^ ^,^ ^97^ ^,^ ^98^ ^,^ ^99^ ^,^ ^101^ ^,^ ^102^ ^,^ ^104^ ^,^ ^105^ ^,^ ^106^ ^,^ ^111^.

Quantitative cross-sectional studies are also observed (30%), which analyze the association of constructs with a focus on risk and protective factors ^14^ ^,^ ^36^ ^,^ ^37^ ^,^ ^38^ ^,^ ^39^ ^,^ ^42^ ^,^ ^43^ ^,^ ^60^ ^,^ ^62^ ^,^ ^65^ ^,^ ^66^ ^,^ ^68^ ^,^ ^70^ ^,^ ^71^ ^,^ ^73^ ^,^ ^74^ ^,^ ^75^ ^,^ ^76^ ^,^ ^77^ ^,^ ^78^ ^,^ ^82^ ^,^ ^84^ ^,^ ^91^ ^,^ ^100^ ^,^ ^109^ ^,^ ^110^ ^,^ ^112^ ^,^ ^113^ ^,^ ^114^. Others list strategies and programs with strong scientific evidence and effectiveness testing ^40^ ^,^ ^54^ ^,^ ^56^ ^,^ ^115^ ^,^ ^116^. One study presents a validation study of a scale on emotion regulation challenges ^45^. Interventions are analyzed in a school, community or clinical context ^63^ ^,^ ^83^ ^,^ ^85^ ^,^ ^89^ ^,^ ^108^.

The actions assess the application of suicide/self-harm prevention strategies/programs among adolescents aiming to strengthen socioemotional skills ^13^ ^,^ ^57^ ^,^ ^70^, implement actions for adolescents at risk ^61^, support teacher training to detect suicidal/self-harm behavior ^57^ ^,^ ^91^, and adopt treatment-based interventions with an emphasis on dialectical behavior therapy ^114^.

Interventions based on emotional intelligence with adolescents presenting a history of suicidal/self-harm behavior ^83^ ^,^ ^85^ ^,^ ^89^ ^,^ ^108^, especially in school and community contexts ^87^ ^,^ ^106^ tend to produce positive effects. However, few of them use methods that show robust evidence of results ^11^ ^,^ ^86^. The strategies analyzed in studies are based on revealing emotions and/or feelings, resolving conflicts, and finding alternative solutions in the face of adversity. They include social interaction skills, which require empathy, reciprocity, cooperation, and negotiation strategies, as well as good relationships ^44^ ^,^ ^53^ ^,^ ^57^ ^,^ ^69^ ^,^ ^80^. With these skills, adolescents would be better prepared to address daily social and individual challenges and risk factors associated with suicidal/self-harm behavior ^60^ ^,^ ^64^ ^,^ ^74^ ^,^ ^83^ ^,^ ^117^.

### The role of school

There is a consensus on the role of school as an important place to address emotions in a strategy to prevent suicidal behavior and self-harm ^52^. With more or less emphasis, the studies reinforce the need to include the emotional dimension as an essential element for cognitive development, responsible for controlling feelings and emotions and indispensable in selecting information to guide thinking and actions in social and cultural practices ^35^ ^,^ ^48^ ^,^ ^54^ ^,^ ^85^ ^,^ ^99^. Schools are strategic for the development of universal prevention actions, i.e. those for all audiences aiming to help them acquire skills of acceptance and emotional tolerance, promoting a school environment of emotional validation ^35^ ^,^ ^46^ ^,^ ^57^ ^,^ ^76^ ^,^ ^87^ ^,^ ^99^. Selective prevention programs are also indicated for those who already show warning signs of suicidal or self-harm behavior ^35^ ^,^ ^101^ ^,^ ^115^, helping adolescents deal with the negative emotional states resulting from interpersonal difficulties and avoid their negative impact on mental health ^46^ ^,^ ^76^ ^,^ ^87^ ^,^ ^99^ ^,^ ^106^.

Actions for parents and teachers are essential for the development of their own socioemotional skills and those of adolescents ^7^ ^,^ ^92^. In the perspective of psychoeducation, the studies report that parents and teachers must be informed about the negative effects of interpersonal relationships that involve threat, criticism, subordination, and depreciation.

The studies emphasize the positive effects of the school environment, the importance of developing listening spaces and encouraging mechanisms for students to develop socioemotional skills ^51^ ^,^ ^53^. In general, the interventions at school are based on a standard curriculum and adapted to the school and sociocultural reality. Longer curricula (not necessarily in hours, but over time) seem to be more manageable, as those that train school staff and teachers. Actions in the school environment reduce violence, improve learning, increase the time of adolescents at school, facilitate peer relationships, and reduce suffering - important elements for well-being and, consequently, for the prevention of suicidal behavior.

Among the actions highlighted in the school context is training the education team to act as guardians, creating a supportive school environment, recognizing risk factors and warning signs of suicidal behavior, supporting distressed students, and enabling collaborative actions to obtain additional support for those in distress ^92^ ^,^ ^115^. In addition, they have a focus on promoting staff mental health (training and access to support) ^2^ ^,^ ^52^ ^,^ ^92^ and training on the healthy use of the internet and social media ^51^. They also encourage a safe school environment, with anti-bullying programs and initiatives to strengthen social connections ^92^ ^,^ ^93^ ^,^ ^112^. Other important actions include creating, strengthening, and advertising contacts with external support services and providing clear policy and protocols for staff when a risk of suicide is identified. In addition, it is essential to support the return to school for a student after a suicide attempt ^92^ ^,^ ^96^. Parents should be engaged in this process to raise awareness of mental health ^57^ ^,^ ^82^. The importance of early discussion about suicidal behavior and stress management is highlighted, encouraging emotion regulation and anti-stigma actions ^88^. Earlier prevention and intervention tend to produce better outcomes. Another recommendation is to create spaces where children, adolescents, and parents/guardians can find help, advice, information, and online tools ^74^.

### Implementation of actions in health services

Strategies in health seem to result from actions in education, highlighting the role of intersectorality, showing a scenario in which those who “did not work out as a result of school actions” come to a health service already in a serious situation; i.e. preventive actions in health would be selective or recommended, while school actions would be basically universal. It is important to bring the health system into basic education. In terms of implementation in the Brazilian Unified National Health System (SUS, acronym in Portuguese), care strategies must not lose sight of the principles of health promotion and primary health care ^3^ ^,^ ^98^ ^,^ ^99^ ^,^ ^102^, while identifying warning signs so that young people can have alternative coping actions available to them in mental health programs and support groups. These initiatives can involve schools, which could provide training to guardians, peers, and adults on how to recognize warning signs of suicide. They can also develop a school culture of psychological well-being and screening to identify those who may be at risk ^67^ ^,^ ^81^ ^,^ ^85^ ^,^ ^101^.

The main strategies for primary health care are: (i) promotion of health in the community, ensuring the right to comprehensive health from universal access to services that promote equity and effective coverage; (ii) guarantee of comprehensive, integrated, appropriate, quality care that is sustained over time; (iii) development of mechanisms for intersectoral articulation and participation of all community actors in the planning and development of interventions; and (iv) creation of participatory planning and implementation methodologies aiming to identify prevalent problems in the territory, map actors and resources, address emergency conditions, detect and enhance the protective factors of individuals in their singularity and of communities, and evaluate processes and results ^49^ ^,^ ^51^ ^,^ ^102^.

The studies also discuss the need for a cross-sectional approach at all levels of the health system. It seems essential to have a strategic positioning of teams at the first level of care, implementing a role of mediation and coordination that effectively transforms the pyramid of care into a network, where relationships between the health team and other services and institutions are based on cooperative actions ^54^ ^,^ ^77^ ^,^ ^96^ ^,^ ^102^. It is crucial to prioritize and implement intra- and inter-institutional interventions according to the reality of each territory and the personal and community stories, which must be settled in order to restore and strengthen the social bond ^54^ ^,^ ^102^ ^,^ ^118^.

### Governmental and nongovernmental actions and the role of the media

Governmental actions are crucial for the prevention of suicidal/self-harm behavior because of the potential to create and implement regulations at municipal, state, and federal levels, such as restriction to lethal means. In addition, interventions with the participation of society as a whole and institutions for children and adolescents are relevant, including workshops; forums; round tables with teachers, parents/guardians, and children/adolescents; articulation of governmental bodies from municipal, state, and federal levels with schools, youth organizations, clubs, recreational centers, nongovernmental organizations (NGOs), etc.; studies to analyze strategic themes at the local level; identification and training of adolescent tutors/leaders in their communities; promotion of processes of knowledge transfer and peer learning; dissemination of cultural, recreational, and educational resources for children and adolescents; implementation of mental health advisory services in schools; and prevention and awareness campaigns in the media and public spaces ^77^ ^,^ ^102^ ^,^ ^104^ ^,^ ^116^.

Media were highlighted in a significant number of studies as a partner in the educational prevention process. They are also indicated as a strategic place in the debate and promotion of actions related to communication rules in the media about suicidal events ^2^ ^,^ ^77^ ^,^ ^95^. Emphasis is placed on media role in raising awareness and reducing the stigma of suicide, in the sense of restricting information about the means and environments/places that favor the act of suicide, without giving visibility to methods of suicide or self-harm. It is suggested that media can help by disseminating information about where to seek help and can help develop guidelines for responsible coverage of suicide, training professionals in reporting cases. Developing policies to monitor content on digital media platforms, creating web pages designed to help young people manage or reduce suicidal ideation or self-harm, and promoting social interaction by enabling peer support are other actions cited in the literature ^2^ ^,^ ^3^ ^,^ ^51^ ^,^ ^93^ ^,^ ^99^ ^,^ ^102^ ^,^ ^119^.

## Discussion

In Brazil and around the world, little is known about what can be done to prevent suicidal behavior and self-harm. Although different strategies have been described, few of them have been clearly tested and validated. Health services, schools, and social protection services face many challenges and impasses when handling children and adolescents who deliberately hurt themselves, and think about or try to kill themselves. Our scoping review shows that: (i most existing initiatives are international; (ii) most knowledge produced is focused on suicide, few exclusively address self-harm, and others address both, with little emphasis on their differences; (iii) schools are key actors in developing preventive actions, and health has to expand its actions and act in a collaborative network; and (iv) the main themes developed in prevention actions around the world focus on early identification of suicidal behavior/self-harm, promotion of socioemotional life, restricted access to means, media support, and a focus on mental health.

This scoping review reveals that actions focused on socioemotional skills are strategic, as they support the process of expression and production of emotions, acting on the meaning of the situation and modulating the emotional response. They involve physiological, cognitive, behavioral, and experiential components with varied intensity and subjective evaluation by the individual, and can usually be triggered by interpersonal situations and events that deserve attention because they affect well-being ^120^. This way, adolescents change the way they expose themselves to certain situations, with the ability to better assess a problem, reducing tension and presenting a more flexible cognitive and emotional response to events ^42^ ^,^ ^118^ ^,^ ^120^.

Also important are the prevention actions based on socioemotional skills with a focus on parents and teachers ^7^ ^,^ ^92^. In addition, intersectoral collaboration with governmental and nongovernmental actors is essential for early care, monitoring, and evaluation of cases ^2^ ^,^ ^3^. The fields of health, education, and social care should act together, since they are strategic in the daily care of children and adolescents with early or already established signs of suicidal behavior/self-harm. Significant actions have been proposed in a debate seeking to create protective environments by reducing access to lethal means. In Brazil, the National Policy for the Prevention of Self-Harm and Suicide, through *Law n. 13,819/2019* ^121^, represents an important legal framework and emphasizes the need for prevention strategies, but does not guarantee advances in mental health care.

In general, prevention programs provide tips and show how to deal with situations. Few of them detail the implementation of their actions and often fail to present results and evaluation criteria. Therefore, it is strongly recommended that actions and results should be documented. Another weakness concerns the debate and primary findings on specific prevention actions for vulnerable groups, such as the LGBTQIA+ population, unemployed people, migrants, people deprived of liberty or with black skin, which are aspects with timid discussion in the studies.

One of the strengths of this review is the breadth of the bibliographic search, which included literature from strategic sources, ensuring a new character to this review as well as an emphasis on prevention based on socioemotional aspects.

Finally, in many parts of the world, and especially in Brazil, there are still no clear guidelines for the prevention of suicidal and self-harm behavior. The government has to assume a leading role in guiding the care and protection of children, adolescents, and their family members who are suffering. Brazil has not yet made progress in creating national and local prevention plans, with different guidelines for each of these behaviors and the adoption of a practical and effective approach, emphasizing the role of the SUS, mental health care, and intersectoral coordination for the promotion of health and quality of life and prevention of suffering and mental disorders.
