# Supplementary material for: Scoping review on socioemotional skills in the prevention of suicidal behavior among adolescents
Source: Cad Saude Publica. 2024 Aug 26;40(7):e00002524. doi: 10.1590/0102-311XEN002524 (PMC11349282; doi:10.1590/0102-311XEN002524)
Supplement: Translation

# Translation: Revisão de escopo sobre habilidades socioemocionais na prevenção do comportamento suicida em adolescentes

**DOI:** 10.1590/0102-311XPT002524

**Authors:** Joviana Quintes Avanci, Aline Ferreira Gonçalves, Orli Carvalho da Silva, Pedro Henrique Tavares, Simone Gonçalves de Assis

**Affiliations:** 1 Escola Nacional de Saúde Pública Sergio Arouca, Fundação Oswaldo Cruz, Rio de Janeiro, Brasil.; 2 Instituto Nacional de Saúde da Mulher, da Criança e do Adolescente Fernandes Figueira, Fundação Oswaldo Cruz, Rio de Janeiro, Brasil.

**Keywords:** Suicídio, Autolesão, Ideação Suicida, Adolescente, Prevenção do Suicídio

## Abstract

Promover habilidades socioemocionais tem sido destacado, entre as evidências, como prevenção do comportamento suicida na infância e na adolescência. Este artigo visa mapear e analisar a produção científica nacional e internacional sobre iniciativas e programas de prevenção do comportamento suicida na adolescência baseados no referencial teórico das habilidades socioemocionais. Caracteriza-se por uma revisão de escopo utilizando a metodologia proposta pelo Instituto Joanna Briggs. Foram consultadas 11 bases bibliográficas acadêmicas, além de busca em *sites* institucionais relacionados à prevenção de suicídio e no Google. Foram incluídas publicações em português, espanhol, francês e inglês entre os anos de 2010 a julho de 2022. O acervo foi composto por 97 publicações, analisadas por meio da matriz de dados e agrupamento temático. Os resultados mostram que a maioria das iniciativas é internacional e voltada para o suicídio, sem privilegiar a autolesão. De forma geral, apresentam viés informativo e instrucional voltado para profissionais, instituições e governos, projetos de lei, programas e planos de ação, estudos sobre o papel das competências socioemocionais e pesquisas de intervenção. Poucas estratégias são claramente testadas e validadas. Os elementos-chave são a capacidade para perceber, reconhecer, compreender, expressar e regular as próprias emoções, motivar-se e estabelecer relações de empatia. As escolas são protagonistas e a saúde precisa atuar em rede colaborativa. São necessários planos nacionais e locais de prevenção, enfatizando o papel da escola, do setor saúde e da articulação intersetorial para a promoção de saúde e qualidade de vida.

## Introdução

Promover habilidades socioemocionais tem sido destacado como uma forma de prevenção do comportamento suicida na infância e adolescência, ao lado da limitação ao acesso aos meios de suicídio; da interação com a mídia para a denúncia responsável, a formação e o cuidado; e a identificação e o acompanhamento precoces ^1^ ^,^ ^2^ ^,^ ^3^. Teorias sobre suicídio propõem que adolescentes incapazes de produzir estratégias de como lidar com o afeto negativo têm a avaliação cognitiva impactada, o que leva a pensamentos ou expectativas negativas sobre o futuro e a percepção do ato suicida como única saída ^4^ ^,^ ^5^. Ademais, a falta de conectividade social e de pertencimento, a existência de sentimentos de derrota e aprisionamento dominam o estado emocional. Assim, a falta de consciência e compreensão das emoções, a incapacidade de controlar comportamentos impulsivos e de utilizar estratégias resolutivas, e a existência da dor combinada com a desesperança podem ser condições para o desenvolvimento do quadro suicida ^6^.

Desse modo, muitas intervenções promissoras, com famílias, escolas ou em serviços de saúde, partem da atuação nas habilidades socioemocionais, priorizando a regulação das emoções, a resolução de problemas e a ampliação da capacidade relacional ^1^ ^,^ ^7^. Essas habilidades agregam aspectos socioafetivos, emocionais, comportamentais e morais. Entre os constructos teóricos estão a inteligência e a regulação emocional, podendo ser fatores protetores diante de instabilidade emocional, dificuldade de adaptação, culpa, sentimentos de fracasso, frustração, medo e impulsividade ^8^ ^,^ ^9^ ^,^ ^10^ ^,^ ^11^ ^,^ ^12^. São passíveis de intervenções e podem ser ensinadas, aprendidas e praticadas ao longo da vida, visando uma sensação de bem-estar e de melhor interação social. O déficit na habilidade social e de regular as próprias emoções parece ser precursor do comportamento suicida, em que a busca pelo ato pode propiciar uma sensação falsa de alívio ^13^ ^,^ ^14^.

Conceitualmente, o comportamento suicida se refere a um tipo de conduta da pessoa que busca se ferir ou se matar ^15^. Aparece sob a forma de ideação, planejamento ou tentativa suicida, além da autoagressão, que pode não estar relacionada à intencionalidade suicida ^15^. Em geral, as fronteiras entre essas ações são tênues ^14^ ^,^ ^16^ ^,^ ^17^. As taxas de suicídio na adolescência são preocupantes, sendo a quarta causa de morte entre jovens de 15 a 29 anos no mundo ^18^. No Brasil, há tendência ascendente do suicídio a partir do ano 2000 ^19^ ^,^ ^20^. Sexo, idade e raça/etnia têm implicações importantes na epidemiologia do suicídio, principalmente, quando predispõem contextos de vulnerabilidades sociais, discriminação e violência ^21^. É um fenômeno multifatorial, com a interação de fatores biológicos, sociais, psicológicos e filosófico-existenciais ^22^. As redes sociais virtuais têm recebido destaque nesse debate por propiciar risco e proteção ^23^.

A partir da ascensão do problema no Brasil e em partes do mundo, especialmente na adolescência, a Organização Mundial da Saúde (OMS) e órgãos nacionais têm sido convocados a delinear estratégias de prevenção para garantir acesso aos serviços públicos de saúde, fortalecer políticas e financiamentos a locais e populações vulneráveis, e oferecer tratamento integral, com ênfase em ações preventivas ^13^ ^,^ ^14^ ^,^ ^16^ ^,^ ^24^ ^,^ ^25^ ^,^ ^26^ ^,^ ^27^ ^,^ ^28^ ^,^ ^29^. Diante da relevância dos aspectos socioemocionais, esse estudo visa mapear e analisar a produção científica nacional e internacional a respeito de iniciativas e programas sobre prevenção do comportamento suicida na adolescência baseados no referencial teórico das habilidades socioemocionais, a fim de subsidiar ações que possam ser implementadas na educação, na saúde, em serviços governamentais, não governamentais e na mídia.

## Método

### Delineamento do estudo

Caracteriza-se por uma revisão de escopo, método sistemático para o mapeamento da produção científica sobre determinado tema com o objetivo de identificar conceitos e lacunas. O referencial metodológico baseou-se no manual do Instituto Joanna Briggs ^30^ e incluiu publicações de métodos de pesquisa e fontes documentais variadas. A seguinte pergunta orientou a revisão: como as estratégias e programas sobre habilidades socioemocionais são desenvolvidas para a prevenção do comportamento suicida na infância e na adolescência? Um protocolo da revisão foi elaborado e registrado no Open Science Framework (OSF) ^31^ e seguiu as diretrizes do PRISMA (*Preferred Reporting Items for Systematic Reviews and Meta-Analyses*) ^32^.

### Estratégias de busca

As buscas foram efetuadas entre janeiro e julho de 2022 e englobaram duas etapas organizadas segundo tipo de acervo: (i) voltada para bases acadêmicas, que incluiu 11 bases bibliográficas - SciELO, Portal Regional da BVS, Portal Regional da BVS (Descritores em Ciências de Saúde; DeCS), OASIS (do Instituto Brasileiro de Informação em Ciência e Tecnologia; IBICT), Scopus, Web of Science, PubMed/MEDLINE (título e resumo), PubMed (termos do *Medical Subject Headings*; MeSH), Dimensions, Embase (descritores Emtree) e PsycNET - e Google Acadêmico; e (ii) busca em *sites* institucionais relacionados à prevenção de suicídio e no Google.

A busca estruturada foi adaptada para cada base investigada e tipo de acervo por um bibliotecário experiente a partir dos seguintes descritores: “prevenção”, “suicídio” (“comportamento suicida” OR “ideação suicida” OR “suicídio” OR “autolesão” OR “comportamento autodestrutivo), “inteligência emocional” (“manejo emocional” OR “regulação emocional” OR “controle emocional” OR “autocontrole emocional” OR “gerenciamento emocional), “adolescente” e “criança” (Quadro 1). Foram incluídas publicações no idioma português, espanhol, francês e inglês, tendo como referência temporal os anos de 2010 a julho de 2022. Estudos primários, secundários, relatos de experiência, ensaios teóricos, teses, dissertações, documentos oficiais, relatórios, entre outros, fizeram parte da pesquisa.

**Quadro 1:** Descrição das bases bibliográficas utilizadas segundo chaves de busca e número de publicações encontradas, 2010-2022.

BASES BIBLIOGRÁFICAS	CHAVES DE BUSCA	PUBLICAÇÕES
SciELO: *site* com periódicos de acesso gratuito. Busca integrada de artigos dos periódicos da rede SciELO: Argentina, Brasil, Chile, Colômbia, Cuba, Espanha, Portugal, Venezuela, Saúde Pública e Ciências Sociais. A SciELO é uma biblioteca eletrônica que abrange uma coleção selecionada de periódicos científicos. A SciELO é o resultado de um projeto de pesquisa da Fundação de Amparo à Pesquisa do Estado de São Paulo (FAPESP), em parceria com o Centro Latino-Americano e do Caribe de Informação em Ciências da Saúde (BIREME).	(“inteligência emocional” OR “manejo emocional” OR “regulação emocional” OR “controle emocional” OR “autocontrole emocional” OR “Gerenciamento emocional” OR “Emotional Intelligence” OR “Emotional Intelligences” OR “Intelligence, Emotional” OR “Intelligence, Social” OR “Intelligences, Emotional” OR “Intelligences, Social” OR “Social Intelligence” OR “Social Intelligences”) AND (“comportamento suicida” OR “ideação suicida” OR suicídio OR Suicidal OR “ehavior de suicídio” OR autolesão OR “Comportamento Autodestrutivo” OR “Self-Injurious Behavior” OR “Conducta Autodestructiva” OR “Deliberate Self Harm” OR “Deliberate Self-Harm” OR “Harm, Self” OR “Intentional Self Harm” OR “Intentional Self Injuries” OR “Intentional Self Injury” OR “Non Suicidal Self Injury” OR “Non-Suicidal Self Injuries” OR “Non-Suicidal Self Injury” OR “Nonsuicidal Self Injuries” OR “Nonsuicidal Self Injury” OR “Self Destructive Behavior” OR “Self Harm” OR “Self Harm, Intentional” OR “Self Injuries, Non-Suicidal” OR “Self Injuries, Nonsuicidal” OR “Self Injurious Behavior” OR “Self Injury” OR “Self Injury, Intentional” OR “Self Injury, Non-Suicidal” OR “Self Injury, Nonsuicidal” OR “Self-Destructive Behavior” OR “Self-Destructive Behaviors” OR “Self-Harm, Deliberate” OR “Self-Injuries” OR “Self-Injurious Behaviors” OR “Self-Injury” OR “Autoagressão Intencional” OR “Conduta Autolesiva” OR “Ferimento Autoinfligido não Suicida” OR “Lesão Autoinfligida não Suicida”)) AND (Adolesc* OR adolescência OR Teenager) AND (Prevenção OR Prevention OR Prevención)	0
Portal Regional da BVS (título, resumo e assunto). Busca integrada nas bases de dados da BIREME: LILACS - Literatura Latino-Americana e do Caribe em Ciências da Saúde MEDLINE - Literatura Internacional em Ciências da Saúde ADOLEC - Saúde na Adolescência ADSAUDE - Administração de Serviços de Saúde BBO - Bibliografia Brasileira de Odontologia BDNEF - Base de dados de Enfermagem BIOÉTICA - Base de dados do Programa Regional de Bioética da Organização Pan-Americana da Saúde (OPAS)/Organização Mundial da Saúde (OMS) DESASTRES - Acervo do Centro de Documentação de Desastres HISA - História da Saúde Pública na América Latina e Caribe HOMEOINDEX - Bibliografia Brasileira de Homeopatia LEYES - Legislação Básica de Saúde da América Latina e Caribe MECARIB - Literatura do Caribe em Ciências da Saúde REPIDISCA - Literatura em Engenharia Sanitária e Ciências do Ambiente PAHO - Acervo da Biblioteca da Organização Pan-Americana da Saúde WHOLIS - Sistema de Informação da Biblioteca da OMS	(“inteligência emocional” OR “manejo emocional” OR “regulação emocional” OR “controle emocional” OR “autocontrole emocional” OR “Gerenciamento emocional” OR “Emotional Intelligence” OR “Emotional Intelligences” OR “Intelligence, Emotional” OR “Intelligence, Social” OR “Intelligences, Emotional” OR “Intelligences, Social” OR “Social Intelligence” OR “Social Intelligences”) AND (“comportamento suicida” OR “ideação suicida” OR suicídio OR suicidal OR “ehavior de suicídio” OR autolesão OR “Comportamento Autodestrutivo” OR “Self-Injurious Behavior” OR “Conducta Autodestructiva” OR “Deliberate Self Harm” OR “Deliberate Self-Harm” OR “Harm, Self” OR “Intentional Self Harm” OR “Intentional Self Injuries” OR “Intentional Self Injury” OR “Non Suicidal Self Injury” OR “Non-Suicidal Self Injuries” OR “Non-Suicidal Self Injury” OR “Nonsuicidal Self Injuries” OR “Nonsuicidal Self Injury” OR “Self Destructive Behavior” OR “Self Harm” OR “Self Harm, Intentional” OR “Self Injuries, Non-Suicidal” OR “Self Injuries, Nonsuicidal” OR “Self Injurious Behavior” OR “Self Injury” OR “Self Injury, Intentional” OR “Self Injury, Non-Suicidal” OR “Self Injury, Nonsuicidal” OR “Self-Destructive Behavior” OR “Self-Destructive Behaviors” OR “Self-Harm, Deliberate” OR “Self-Injuries” OR “Self-Injurious Behaviors” OR “Self-Injury” OR “Autoagressão Intencional” OR “Conduta Autolesiva” OR “Ferimento Autoinfligido não Suicida” OR “Lesão Autoinfligida não Suicida”) AND (adolesc* OR adolescência OR teenager OR Criança* OR Niños OR Child OR Chiildhood OR Infancy) AND (prevenção OR prevention OR prevención) AND (year_cluster:[2010 TO 2022])	30
Portal Regional da BVS (Descritores em Ciências da Saúde - DeCS) (idem acima)	(e☹ “Inteligência Emocional” OR )) AND (e☹Suicídio OR “Comportamento Autodestrutivo” OR “Tentativa de suicídio”)) AND (e☹ Adolescen* OR Crianças)) AND (e☹ Prevenção OR Preventivo))	0
OASIS (Instituto Brasileiro de Informação em Ciência e Tecnologia - IBICT): portal brasileiro de repositórios e periódicos de acesso aberto. Permite, por meio de uma única interface, a pesquisa simultânea em todos os repositórios digitais e periódicos científicos eletrônicos que utilizam o Protocolo OAI-PMH, constituindo-se, nesse sentido, em um provedor de serviços. Ou seja, os provedores de dados (instituições, periódicos científicos) expõem os metadados que descrevem os seus conteúdos para que sejam coletados pelo provedor de serviços que centraliza serviços de buscas. O IBICT desenvolveu e coordena a Biblioteca Digital Brasileira de Teses e Dissertações (BDTD), que integra os sistemas de informação de teses e dissertações existentes nas instituições de ensino e pesquisa do Brasil, e também estimula o registro e a publicação de teses e dissertações em meio eletrônico. A BDTD, em parceria com as instituições brasileiras de ensino e pesquisa, possibilita que a comunidade brasileira de ciência & tecnologia publique e difunda suas teses e dissertações produzidas no país e no exterior, dando maior visibilidade à produção científica nacional	(“inteligência emocional” OR “manejo emocional” OR “regulação emocional” OR “controle emocional” OR “autocontrole emocional” OR “Gerenciamento emocional” OR “Emotional Intelligence” OR “Emotional Intelligences” OR “Intelligence, Emotional” OR “Intelligence, Social” OR “Intelligences, Emotional” OR “Intelligences, Social” OR “Social Intelligence” OR “Social Intelligences”) AND (“comportamento suicida” OR “ideação suicida” OR suicídio OR Suicidal OR “ehavior de suicídio” OR autolesão OR “Comportamento Autodestrutivo” OR “Self-Injurious Behavior” OR “Conducta Autodestructiva” OR “Deliberate Self Harm” OR “Deliberate Self-Harm” OR “Harm, Self” OR “Intentional Self Harm” OR “Intentional Self Injuries” OR “Intentional Self Injury” OR “Non Suicidal Self Injury” OR “Non-Suicidal Self Injuries” OR “Non-Suicidal Self Injury” OR “Nonsuicidal Self Injuries” OR “Nonsuicidal Self Injury” OR “Self Destructive Behavior” OR “Self Harm” OR “Self Harm, Intentional” OR “Self Injuries, Non-Suicidal” OR “Self Injuries, Nonsuicidal” OR “Self Injurious Behavior” OR “Self Injury” OR “Self Injury, Intentional” OR “Self Injury, Non-Suicidal” OR “Self Injury, Nonsuicidal” OR “Self-Destructive Behavior” OR “Self-Destructive Behaviors” OR “Self-Harm, Deliberate” OR “Self-Injuries” OR “Self-Injurious Behaviors” OR “Self-Injury” OR “Autoagressão Intencional” OR “Conduta Autolesiva” OR “Ferimento Autoinfligido não Suicida” OR “Lesão Autoinfligida não Suicida”) AND (Adolesc* OR adolescência OR Teenager OR Criança* OR Niños OR Child OR Chiildhood OR Infancy) AND (Prevenção OR Prevention OR Prevención)	4
Scopus: referênciais com resumos. Scopus é um banco de dados científico, médico, técnico e de ciências sociais abrangente que contém toda a literatura relevante	(TITLE-ABS-KEY (“Emotional Intelligence” OR “Emotional Intelligences” OR “Intelligence, Emotional” OR “Intelligence, Social” OR “Intelligences, Emotional” OR “Intelligences, Social” OR “Social Intelligence” OR “Social Intelligences”) AND TITLE-ABS-KEY (suicidal OR “Suicide ideation” OR “Self-Injurious Behavior” OR “Behavior, Self-Destructive” OR “Behavior, Self-Injurious” OR “Behaviors, Self-Destructive” OR “Behaviors, Self-Injurious” OR “Deliberate Self Harm” OR “Deliberate Self-Harm” OR “Harm, Self” OR “Intentional Self Harm” OR “Intentional Self Injuries” OR “Intentional Self Injury” OR “Non Suicidal Self Injury” OR “Non-Suicidal Self Injuries” OR “Non-Suicidal Self Injury” OR “Nonsuicidal Self Injuries” OR “Nonsuicidal Self Injury” OR “Self Destructive Behavior” OR “Self Harm” OR “Self Harm, Intentional” OR “Self Injuries, Non-Suicidal” OR “Self Injuries, Nonsuicidal” OR “Self Injurious Behavior” OR “Self Injury” OR “Self Injury, Intentional” OR “Self Injury, Non-Suicidal” OR “Self Injury, Nonsuicidal” OR “Self-Destructive Behavior” OR “Self-Destructive Behaviors” OR “Self-Harm, Deliberate” OR “Self-Injuries” OR “Self-Injurious Behaviors” OR “Self-Injury”) AND TITLE-ABS-KEY (adolesc* OR teenager OR child OR infancy OR kids OR children) AND TITLE-ABS-KEY (prevention))	12
Web of Science: base multidisciplinar que indexa somente os periódicos mais citados em suas respectivas áreas. É também um índice de citações, informando, para cada artigo, os documentos por ele citados e os documentos que o citaram. Contém hoje mais de 9.000 periódicos indexados. É composta por Science Citation Index Expanded (SCI-EXPANDED): 1945 até o presente; Social Sciences Citation Index: 1956 até o presente; Arts and Humanities Citation Index: 1975 até o presente. A partir de 2012, o conteúdo foi ampliado com a inclusão do Conference Proceedings Citation Index - Science (CPCI-S); Conference Proceedings Citation Index - Social Science & Humanities (CPCI-SSH)	“Emotional Intelligence” OR “Emotional Intelligences” OR “Intelligence, Emotional” OR “Intelligence, Social” OR “Intelligences, Emotional” OR “Intelligences, Social” OR “Social Intelligence” OR “Social Intelligences” (Todos os campos) and Suicidal OR “Suicide ideation” OR “Self-Injurious Behavior” OR “Behavior, Self-Destructive” OR “Behavior, Self-Injurious” OR “Behaviors, Self-Destructive” OR “Behaviors, Self-Injurious” OR “Deliberate Self Harm” OR “Deliberate Self-Harm” OR “Harm, Self” OR “Intentional Self Harm” OR “Intentional Self Injuries” OR “Intentional Self Injury” OR “Non Suicidal Self Injury” OR “Non-Suicidal Self Injuries” OR “Non-Suicidal Self Injury” OR “Nonsuicidal Self Injuries” OR “Nonsuicidal Self Injury” OR “Self Destructive Behavior” OR “Self Harm” OR “Self Harm, Intentional” OR “Self Injuries, Non-Suicidal” OR “Self Injuries, Nonsuicidal” OR “Self Injurious Behavior” OR “Self Injury” OR “Self Injury, Intentional” OR “Self Injury, Non-Suicidal” OR “Self Injury, Nonsuicidal” OR “Self-Destructive Behavior” OR “Self-Destructive Behaviors” OR “Self-Harm, Deliberate” OR “Self-Injuries” OR “Self-Injurious Behaviors” OR “Self-Injury” (Todos os campos) and Adolesc* OR Teenager OR child OR infancy OR kids OR children (Todos os campos) and Prevention (Todos os campos).	11
PubMed/MEDLINE (título e resumo): base de dados especializada em ciências biomédicas e ciências da vida. Foi desenvolvida pelo Insituto Nacional de Saúde do Estados Unidos (NIH) e administrada pelo Centro Nacional de Informação Biotecnológica (NCBI). De acesso público, indexa a literatura especializada nas áreas de ciências biológicas, enfermagem, odontologia, medicina, medicina veterinária e saúde pública	(((((“emotional intelligence”[Title/Abstract]) OR (“social intelligence”[Title/Abstract])) OR (Intelligence,[Title/Abstract])) AND (((“suicidal behavior”[Title/Abstract]) OR (“suicide”[Title/Abstract])) OR (“self destructive behavior”[Title/Abstract]))) AND ((((“adolescence”[Title/Abstract]) OR (“adolescent”[Title/Abstract])) OR (“child”[Title/Abstract])) OR (“kids”[Title/Abstract]))) AND (“prevention”[Title/Abstract])	8
PubMed (termos do *Medical Subject Headings* - MeSH): (idem acima)	(((“emotional intelligence”[MeSH Terms] AND (2010/1/1:2022/5/28[pdat])) AND ((“self injurious behavior”[MeSH Terms]) OR (“suicide”[MeSH Terms]) AND (2010/1/1:2022/5/28[pdat]))) AND ((“adolescent”[MeSH Terms]) OR (“child”[MeSH Terms]) AND (2010/1/1:2022/5/28[pdat]))) AND (prevention AND (2010/1/1:2022/5/28[pdat]))	96
Dimensions: base de dados que oferece coleções abrangentes de dados vinculados em uma plataforma única, desde subsídios, publicações, conjuntos de dados e ensaios clínicos, até patentes e documentos de políticas. Mapeia o ciclo de vida da pesquisa, desde o financiamento ao resultado e seus impactos	(“Emotional Intelligence” OR “Social intelligence”) AND (“Self destructive behavion” OR “Self injuries” OR “Self injurious behavior”) OR (Suicide OR Suicidal) OR (Adolescent Or Adolescence OR Adolescents OR Child OR Childhood OR Kids OR Children OR infancy) AND (Prevention OR Preventive).	24
Embase (descritores Emtree): considerada referência na área de respostas biomédicas e farmacológicas. Em seu *website*, são apresentados os benefícios que a utilização da ferramenta para as áreas de: medicina baseada em evidências, na qual contribui para a tomada de decisões clínicas baseadas em evidências, melhorando os resultados dos pacientes, aumentando a descoberta de evidências biomédicas e fornecendo informações biomédicas abrangentes e atualizadas; farmacovigilância, em que contribui para o monitoramento da literatura da área; desenvolvimento de dispositivos médicos e vigilância pós-comercialização, na qual contribui nas etapas de desenvolvimento de dispositivos médicos com informações biomédicas de alta qualidade, que vai do conceito e design até a vigilância pós-comercialização; desenvolvimento de medicamentos, em que contribui na descoberta de relações entre drogas, doenças e interações medicamentosas, fornecendo informações biomédicas críticas para o desenvolvimento, reposicionamento e segurança de medicamentos. A base proporciona estudos de revisão sistemática e integrativa, diretrizes e protocolos clínicos, avaliação de tecnologias em saúde. Todo o processo de revisões sistemáticas e integrativas é delineado por diretrizes reconhecidas internacionalmente. Inclui também mais de 2,3 milhões de resumos de artigos de conferências desde 2009. Contém atualização diária e inclusão anual de mais de 1,5 milhão de artigos. Contudo, um recurso diferenciado da Embase é a PICO Search, que permite a busca com base nos aspectos da estratégia PICO (Paciente, Intervenção, Comparação e Desfecho), metodologia utilizada na prática baseada em evidências (PBE)	‘emotional intelligence’/exp AND (‘suicide’/exp OR ‘self destructive behavior’/exp OR ‘automutilation’/exp OR ‘suicidal behavior’/exp OR ‘suicide attempt’/exp) AND (‘adolescence’/exp OR ‘childhood’/exp OR ‘child’/exp OR ‘adolescent’/exp) AND prevention AND [2010-2022]/py	12
PsycNET: base de dados em psicologia, educação, psiquiatria e ciências sociais. PsycINFO é a principal base de dados em psicologia existente no mundo, cobrindo praticamente toda a literatura disponível nesse tema. Com atualização semanal, ela oferece milhões de resumos de artigos de periódicos, capítulos de livros, editoriais e demais tipos de referências e citações bibliográficas fornecidos pelas mais conceituadas publicações acadêmicas. Oferece mais de 4 milhões de citações bibliográficas (resumos de artigos). Abrange, atualmente, cerca de 2.500 títulos, sendo 80% do conteúdo oriundo de periódicos (99% dos periódicos disponíveis no PsycINFO são arbitrados, ou seja, passam por revisão por pares). Cobertura completa desde 1880 até o presente, embora contenha registros que datam desde 1597	“Emotional intelligence” OR Abstract: “social intelligence” AND Abstract: “Self injuries” OR Abstract: “Self Injurious behavior” OR Abstract: automultilation OR Abstract: Suicide OR Abstract: Suicidal AND Abstract: Adolesc* OR Abstract: Child* AND Abstract: Prevention OR Abstract: Preventive AND Year: 2010 To 2022	5
TOTAL		202
TOTAL SEM DUPLICIDADE		150

A busca feita no Google foi efetuada em páginas de países com elevadas taxas de mortalidade por suicídio (≥ 2 por 100 mil habitantes) na faixa etária de 0-19 anos, segundo a OMS (2021) (Tabela 1). Foi contabilizado o total de páginas existentes segundo o país selecionado e organizado por regiões mundiais. Os primeiros 20 registros mais relevantes foram analisados a partir do *site* encontrado e/ou de documentos relacionados ao *site* principal. Além disso, foram buscados *sites* institucionais segundo a indicação de especialistas e de informações referidas nos *sites* inicialmente localizados ^33^. Aqui, utilizou-se uma versão simplificada dos termos de busca: inteligência emocional, regulação emocional, habilidade socioemocional, suicídio e autolesão.

**Tabela 1:** Regiões com maiores taxas de mortalidade por suicídio na infância e adolescência e existência de informação sobre inteligência emocional/correlatos e suicídio/autolesão nas páginas Google de cada país.

Região	Países e regiões (OMS)	Países com altas taxas * n (%)	Registros encontrados na 1ª página	Exclusões	Incluídos na planilha final
África	47	27 (57,5)	16	16	0
Américas	33	21 (63,6)	93	78	15
Europa	50	39 (78,0)	101	97	4
Mediterrâneo Oriental	21	8 (38,1)	5	5	0
Pacífico Ocidental	21	11 (52,4)	41	35	6
Sudoeste da Ásia	11	8 (72,7)	19	19	0
Total	183	114 (62,3)	275	250	25

OMS: Organização Mundial da Saúde.

* Países com altas taxas por 100.000 habitantes são considerados aqueles com taxas ≥ 2/100.000 na faixa dos 5-14 anos e/ou > 5,5/100.000 na faixa dos 15-29 anos no ano de 2012.

### Seleção dos estudos e critérios de elegibilidade

A busca inicial ficou com os seguintes números de publicações: (i) 202 publicações em bases bibliográficas acadêmicas; (ii) 300 documentos no Google Acadêmico; (iii) 275 nas páginas do Google dos países com elevadas taxas de suicídio entre crianças e adolescentes; e (iv) 44 em *sites* institucionais.

A partir desse acervo e após a exclusão de duplicatas, a seleção foi feita em duplas a partir de títulos e resumos, utilizando o gerenciador bibliográfico Rayyan (https://www.rayyan.ai/) ^34^. Os avaliadores trabalharam independentemente e o resultado individual foi checado por um terceiro especialista. As divergências foram resolvidas por consenso. Os seguintes critérios de exclusão foram aplicados: ausência da relação entre habilidades socioemocionais e comportamento suicida/autolesão na infância ou adolescência; abordagem de faixa etária que não a infância ou adolescência; abordagem pontual dos temas de interesse; documentos e *sites* não disponíveis; trabalhos sem acesso ao texto completo; *sites* comerciais de venda de livros ou textos; ausência do texto em português, inglês, francês ou espanhol; e produtos audiovisuais, como vídeos e *podcasts*. Foram considerados elegíveis os documentos que versavam sobre os temas das habilidades socioemocionais na relação com o comportamento suicida e a autolesão, com o enfoque da prevenção na infância e adolescência.

A Figura 1 apresenta as etapas de identificação e seleção do acervo, que totalizou 97 publicações. Desse total, 56 são fruto da busca de bases bibliográficas e 41 se referem à junção dos 29 documentos oriundos da busca no Google e 12 da investigação em instituições. A maior parte do acervo de bases não bibliográficas se caracteriza por guia, protocolo ou relatórios governamentais publicados para divulgação científica, seguidos por textos apresentados em *sites*, trabalhos de conclusão de curso, livros, capítulos de livro ou textos estendidos apresentados em congressos, reportagens publicadas *online* e um Projeto de Lei.

**Figura 1:** Etapas da identificação, triagem, elegibilidade e inclusão de documentos no acervo segundo fontes de coleta de informação.

### Análise do acervo

As 97 publicações selecionadas foram analisadas segundo dois movimentos analíticos: (i) caracterização do acervo por meio da matriz de dados com a descrição das publicações segundo título, autoria, resumo, país, ano da publicação, público-alvo, principais conceitos, objetivos e métodos aplicados; e (ii) agrupamento temático segundo ações/estratégias/programas voltados para a escola, serviços de saúde, serviços governamentais e não governamentais e a mídia.

## Resultados

### Caracterização do acervo

No Quadro 2, observa-se que os trabalhos produzidos nos Estados Unidos lideram o acervo analisado (16,5%), ao lado do Brasil e da Espanha (12,3% cada) ^3^ ^,^ ^12^ ^,^ ^35^ ^,^ ^36^ ^,^ ^37^ ^,^ ^38^ ^,^ ^39^ ^,^ ^40^ ^,^ ^41^ ^,^ ^42^ ^,^ ^43^ ^,^ ^44^ ^,^ ^45^ ^,^ ^46^ ^,^ ^47^ ^,^ ^48^ ^,^ ^49^ ^,^ ^50^ ^,^ ^51^ ^,^ ^52^ ^,^ ^53^ ^,^ ^54^ ^,^ ^55^ ^,^ ^56^ ^,^ ^57^ ^,^ ^58^ ^,^ ^59^ ^,^ ^60^ ^,^ ^61^ ^,^ ^62^ ^,^ ^63^ ^,^ ^64^ ^,^ ^65^ ^,^ ^66^ ^,^ ^67^ ^,^ ^68^ ^,^ ^69^ ^,^ ^70^. Em ordem decrescente, estão ainda Colômbia, Austrália, Peru, Argentina, Suíça, Índia, México, Canadá, Portugal, Costa Rica, Reino Unido, Suécia, Nova Zelândia, Países Baixos, Finlândia, Alemanha, Polônia, Bolívia, Equador, Quênia, Malásia, África do Sul, e, por último, Egito, China e Turquia. Quanto ao ano de publicação, a partir de 2012, houve um aumento gradativo de produções, que atingiu o pico em 2018, mantendo uma média de 15 publicações por ano entre os anos de 2018 e 2021.

**Quadro 2:** Categorização do acervo segundo estudo, data de publicação/recuperação do texto, local de produção, atributos socioemocionais e tipo de comportamento suicida/autolesão (n = 97).

ESTUDO	ANO	PAÍS	ATRIBUTOS SOCIOEMOCIONAIS	COMPORTAMENTO SUICIDA/AUTOLESÃO
Buerger et al. ^13^	2022	Alemanha	Regulação emocional	Autolesão
Garmendia Espinoza ^70^	2022	Espanha	Inteligência emocional	Ideação suicida
Schwartz ^122^	2022	Canadá	Regulação emocional	Suicídio
Nova Escola ^50^	2022	Brasil	Inteligência emocional	Suicídio
Hermosillo-de-la-Torre et al. ^84^	2021	México	Regulação emocional, autoestima	Comportamento suicida
Knight ^44^	2021	Estados Unidos	Competências emocionais, atitudes positivas, resiliência etc.	Suicídio
Shahram et al. ^114^	2021	Canadá	Resiliência, *coping*	Suicídio
Massagli et al. ^55^	2021	Brasil	Habilidades socioemocionais	Violência
Rastrollo Sasal ^69^	2021	Espanha	Regulação emocional, habilidades emocionais	Comportamento suicida
Krishnamoorthy & Kalpana ^110^	2021	Índia	Inteligência emocional, habilidades e competências emocionais	Comportamento suicida
Cano Quevedo ^85^	2021	Peru	Saúde emocional	Autolesão
Arguedas González et al. ^123^	2021	Costa Rica	Inteligência emocional, habilidades sociais, autoestima	Suicídio, tentativas, ideação
Organização Mundial da Saúde ^18^	2021	Suíça	Habilidades socioemocionais	Suicídio, autolesão
Organização Mundial da Saúde & Fundo das Nações Unidas para a Infância ^99^	2021	Suíça	Competências emocionais, resiliência	Suicídio, autolesão
Scavacini et al. ^48^	2021	Brasil	Regulação emocional	Autolesão
Farias ^117^	2021	Argentina	Regulação emocional	Autolesão, ideias suicidas
Ministério da Saúde da Argentina et al. ^102^	2021	Argentina	Inteligência emocional, regulação emocional, empatia etc.	Autolesão, suicídio
Ministério da Saúde Pública de Misiones ^111^	2021	Argentina	Inteligência emocional	Suicídio
SOMOS Educação ^53^	2021	Brasil	Inteligência emocional habilidades socioemocionais, resiliência etc.	Suicídio
Rodríguez ^112^	2021	Colômbia	Inteligência emocional, autoestima, empatia	Comportamento suicida
Senado da Colômbia ^104^	2021	Colômbia	Inteligência emocional, empatia e regulação emocional	Suicídio
Conselho Nacional de Políticas Públicas para a Juventude ^96^	2021	Costa Rica	Resiliência	Suicídio
Kim et al. ^14^	2020	Estados Unidos	Regulação emocional	Tentativa de suicídio e autolesão
Acuña de la Cruz & Gamarra Zelada ^82^	2020	Peru	Inteligência emocional	Ideação suicida
Bonet et al. ^63^	2020	Espanha	Inteligência emocional, regulação emocional	Suicídio
Aquino Huanca ^83^	2020	Bolívia	Inteligência emocional	Autolesão
Fernandez Moratilla ^64^	2020	Espanha	Regulação emocional, resiliência, etc.	Suicídio
Arrivillaga et al. ^65^	2020	Espanha	Inteligência emocional	Ideação suicida
Pathare et al. ^90^	2020	Índia	Inteligência emocional	Suicídio
Quintana-Orts et al. ^61^	2020	Espanha	Regulação emocional, inteligência emocional	Ideação suicida
Halicka et al. ^109^	2020	Estados Unidos	Inteligência emocional	Autolesão, comportamento suicida
Velis Giménez ^67^	2020	Espanha	Inteligência emocional, regulação emocional	Autolesão
Fonseca-Pedrero et al. ^68^	2020	Espanha	Regulação emocional, autoestima, habilidades	Comportamento suicida
Magalhães & Carrasco ^56^	2020	Brasil	Habilidades sociais	Comportamento suicida, autolesão
Organização Mundial da Saúde ^106^	2020	Suíça	Resiliência, habilidades emocionais, regulação emocional etc.	Comportamento suicida, autolesão
Trew et al. ^100^	2020	Austrália	Inteligência emocional regulação emocional	Ideação e comportamento suicida
Força-tarefa de Prevenção ao Suicídio da Austrália ^95^	2020	Austrália	Inteligência emocional, regulação emocional, bem-estar emocional	Suicídio
Brausch & Woods ^43^	2019	Estados Unidos	Regulação emocional	Ideação suicida e autolesão
Flores-Kanter et al. ^91^	2019	Espanha	Inteligência emocional, regulação emocional	Ideação suicida
Galarreta Mostacero ^81^	2019	Peru	Regulação emocional, habilidades sociais	Autolesão
Quintana-Orts et al. ^66^	2019	Espanha	Inteligência emocional	Suicídio
Rey et al. ^62^	2019	Espanha	Inteligência emocional	Suicídio
Bezerra ^57^	2019	Brasil	Inteligência emocional, coeficiente emocional	Suicídio
Vollandt ^87^	2019	Estados Unidos	Aprendizagem socioemocional	Suicídio
Scavacini et al. ^47^	2019	Brasil	Regulação emocional	Suicídio, autolesão
Goodman et al. ^124^	2019	SI	Regulação emocional	Autolesão
Força-tarefa de Prevenção ao Suicídio da Austrália ^94^	2019	Austrália	Inteligência emocional	Suicídio
Almeida & Almeida ^54^	2019	Brasil	Inteligência emocional, autoestima, humor positivo	Suicídio
Ministério das Tecnologias da Informação e das Comunicações da Colômbia ^103^	2019	Colômbia	Inteligência emocional	Suicídio
Departamento de Saúde do Território do Norte ^97^	2018/2023	Austrália	Inteligência emocional	Suicídio
Roberts ^41^	2018	Estados Unidos	Resiliência, autorregulação etc.	Ideação suicida
Ganaprakasam ^77^	2018	Malásia	Inteligência emocional	Ideação suicida
Cruz Cob et al. ^116^	2017	México	Autoestima etc.	Comportamento suicida
Gallagher & Miller ^42^	2018	Estados Unidos	Inteligência emocional, autoestima, regulação emocional etc.	Ideação e comportamento suicida
Sánchez ^59^	2018	Espanha	Regulação emocional	Autolesão
Colorado ^118^	2018	Colômbia	Apego e regulação emocional	Comportamento suicida
Okello & Aomo ^78^	2018	Quênia	Inteligência emocional	Comportamento suicida
Zachariah et al. ^79^	2018	Índia	Inteligência e comportamento emocional, regulação emocional	Suicídio
Domínguez-García & Fernández-Berrocal ^60^	2018	Espanha	Inteligência emocional	Comportamento suicida
Fernández ^80^	2018	Equador	Inteligência emocional	Comportamento autodestrutivo
Organização Mundial da Saúde ^92^	2018	Suíça	Regulação emocional	Suicídio
Bloomer ^101^	2018	Reino Unido	Inteligência emocional	Autolesão
Sociedade Brasileira de Neuropsicologia ^49^	2018	Brasil	Regulação emocional, empatia, resiliência	Suicídio
Sucena ^51^	2018	Brasil	Inteligência emocional, bem-estar emocional	Suicídio
Senac Goiás ^52^	2018	Brasil	Inteligência emocional, habilidades socioemocionais, regulação emocional	Suicídio
Organização Mundial da Saúde ^58^	2018	Suíça	Habilidades para a vida, resiliência, regulação emocional	Autolesão, suicídio
Towers Hamlets ^105^	2017/2018	Reino Unido	Inteligência emocional	Suicídio
Alvino Advíncula & Huaytalla Pariona ^74^	2017	Peru	Inteligência emocional	Autolesão
Mohamed et al. ^89^	2017	Egito	Inteligência emocional	Ideação suicida
Du Plooy ^75^	2017	África do Sul	Inteligência emocional	Comportamento suicida
Xavier ^76^	2017	Portugal	Inteligência emocional, regulação emocional	Autolesão
Topper ^40^	2017	Estados Unidos	Inteligência emocional, resiliência etc.	Comportamento suicida
Stone et al. ^3^	2017	Estados Unidos	Regulação emocional	Suicídio
Stern & Divecha ^93^	2017	Finlândia	Inteligência emocional	Suicídio
González Suárez et al. ^113^	2016	Colômbia	Inteligência emocional	Autolesão
Perloe ^38^	2016	Estados Unidos	Inteligência emocional	Autolesão
Kaufman et al. ^45^	2016	Estados Unidos	Regulação emocional	Suicídio, autolesão
Instituto Black Dog ^98^	2016	Austrália	Inteligência emocional	Suicídio
Benito et al. ^108^	2016	Argentina	Inteligência emocional, regulação emocional, empatia etc.	Autolesão e suicídio
Romo et al. ^125^	2016	Suécia	Gestão das emoções	Ideação suicida
Valois et al. ^37^	2015	Estados Unidos	Autoeficácia, reações emocionais	Ideação e tentativa de suicídio
Kwok et al. ^73^	2015	China	Inteligência e competência emocional, resolução de problemas sociais	Ideação suicida
Bodzy et al. ^39^	2015	Estados Unidos	Inteligência emocional, manejo das emoções	Ideação e tentativa de suicídio
Wasserman et al. ^86^	2015	Suécia	Habilidades socioemocionais	Comportamento suicida
Purebl et al. ^2^	2015	Países Baixos	Inteligência emocional, regulação emocional	Suicídio
Fuller et al. ^46^	2015	Estados Unidos	Regulação emocional, habilidades sociais, autoestima	Comportamento suicida, autolesão
Voon et al. ^71^	2014	Austrália	Regulação emocional	Autolesão
Oktan ^72^	2014	Turquia	Autocuidado, autonomia etc.	Autolesão
Santos et al. ^88^	2014	Portugal	Habilidades sociais, autoconceito etc.	Comportamento suicida
Jacobson et al. ^36^	2013	Estados Unidos	Regulação emocional, comunicação social	Tentativa de suicídio
Appelhoff ^115^	2013	Nova Zelândia	Inteligência emocional, bem-estar emocional, resiliência	Suicídio
Awasthi ^126^	2012	Índia	Inteligência emocional	Suicídio
Suárez-Colorado ^127^	2012	Colômbia	Inteligência emocional	Ideação suicida, tentativa e suicídio
Rolston & Lloyd-Richardson ^128^	SD	Estados Unidos	Regulação emocional, *coping*	Autolesão
Departamento de Saúde e Serviços Humanos ^35^	SD	Estados Unidos	Aprendizado socioemocional	Suicídio
Wasserman et al. ^129^	2012	SI	Gerenciamento de emoções	Comportamento suicida
Community-Led Action for Resilience ^107^	SD	Canadá	Resiliência	Suicídio

SD: sem data: SI: sem informação.

A maior parte dos estudos analisados (32%) tem como público-alvo atores da instituição escolar ^13^ ^,^ ^36^ ^,^ ^37^ ^,^ ^44^ ^,^ ^55^ ^,^ ^57^ ^,^ ^61^ ^,^ ^62^ ^,^ ^64^ ^,^ ^65^ ^,^ ^66^ ^,^ ^67^ ^,^ ^68^ ^,^ ^69^ ^,^ ^71^ ^,^ ^72^ ^,^ ^73^ ^,^ ^74^ ^,^ ^75^ ^,^ ^76^ ^,^ ^77^ ^,^ ^78^ ^,^ ^79^ ^,^ ^80^ ^,^ ^81^ ^,^ ^82^ ^,^ ^83^ ^,^ ^84^ ^,^ ^85^ ^,^ ^86^ ^,^ ^87^ ^,^ ^88^. Outras publicações tratam do tema no contexto clínico ^14^ ^,^ ^38^ ^,^ ^39^ ^,^ ^89^, três apresentam uma perspectiva comunitária ^41^ ^,^ ^90^ ^,^ ^91^ e apenas um é de cunho institucional, realizado com crianças sob guarda na Catalunha (Espanha) ^63^. Outro ponto é que a maior parte dos textos se volta para o público adolescente, alguns incluem crianças e adultos jovens ^39^ ^,^ ^92^ ^,^ ^93^ ^,^ ^94^. A abordagem das categorias gênero, orientação sexual, raça/etnia e classe social é restrita à uma breve reflexão teórica, considerada na constituição da amostra e na análise descritiva dos resultados. Contudo, é consenso a relevância do estudo de minorias por sofrerem mais discriminação, isolamento, exclusão ou por terem mais dificuldades no acesso a quaisquer tipos de apoio ^95^. Merecem particular atenção a população LGBTQIA+, jovens de cor de pele preta, comunidades indígenas, rurais, imigrantes e refugiados, adolescentes em privação de liberdade, com deficiência e aqueles em instituições de acolhimento ^2^ ^,^ ^40^ ^,^ ^90^ ^,^ ^94^ ^,^ ^95^ ^,^ ^96^ ^,^ ^97^ ^,^ ^98^ ^,^ ^99^ ^,^ ^100^ ^,^ ^101^.

No que diz respeito aos conceitos pilares das habilidades socioemocionais, há uma base teórica comum nos estudos que parte da aprendizagem socioemocional, regulação emocional, inteligência emocional, inteligência socioemocional, resiliência, empatia, habilidades emocionais, autoconhecimento, competência emocional, promoção da autoestima e saúde mental (Quadro 2). Desses, a inteligência e a regulação emocional se destacam. Os elementos-chave são a capacidade para perceber, reconhecer, compreender, expressar e regular as próprias emoções, motivar-se, reconhecer as emoções das outras pessoas e estabelecer relações de empatia. A expressão das emoções é muito valorizada.

Em geral, as publicações podem ser assim organizadas: (i) aquelas com viés informativo e instrucional voltado para profissionais, instituições e governos, envolvendo não apenas a prevenção do comportamento suicida e da autolesão, mas também hábitos de risco, transtornos mentais, promoção do bem-estar mental, cuidado, enfrentamento de violências e promoção da aprendizagem socioemocional ^47^ ^,^ ^48^ ^,^ ^49^ ^,^ ^94^ ^,^ ^102^ ^,^ ^103^; (ii) Projeto de Lei, o qual toma a educação emocional como um marco integrado ao processo de formação educacional ^104^; (iii) programas e planos de ação ^2^ ^,^ ^3^ ^,^ ^46^ ^,^ ^58^ ^,^ ^95^ ^,^ ^96^ ^,^ ^97^ ^,^ ^98^ ^,^ ^99^ ^,^ ^101^ ^,^ ^102^ ^,^ ^105^ ^,^ ^106^ ^,^ ^107^; (iv) estudos de associação voltados a conhecer o papel das competências socioemocionais no desenvolvimento do comportamento suicida, seja como risco ou proteção ^37^ ^,^ ^38^ ^,^ ^39^ ^,^ ^69^ ^,^ ^73^ ^,^ ^108^ ^,^ ^109^; e (v) pesquisas de intervenção, buscando determinar a eficácia e a aplicabilidade das intervenções, a partir de ações baseadas nas competências socioemocionais e na promoção da saúde mental, na redução do comportamento suicida e da autolesão ^13^ ^,^ ^14^ ^,^ ^57^ ^,^ ^60^ ^,^ ^70^ ^,^ ^86^ ^,^ ^88^ ^,^ ^90^ ^,^ ^110^.

A maior parte dos estudos é teórico (55,6%), com destaque para a prevenção do suicídio e da autolesão (Quadro 2). Também são apontados comportamentos de risco, transtornos mentais, promoção do bem-estar mental, do cuidado, do enfrentamento da violência contra crianças e adolescentes e da aprendizagem socioemocional ^2^ ^,^ ^3^ ^,^ ^46^ ^,^ ^47^ ^,^ ^48^ ^,^ ^49^ ^,^ ^58^ ^,^ ^94^ ^,^ ^95^ ^,^ ^96^ ^,^ ^97^ ^,^ ^98^ ^,^ ^99^ ^,^ ^101^ ^,^ ^102^ ^,^ ^104^ ^,^ ^105^ ^,^ ^106^ ^,^ ^111^.

Estudos quantitativos também têm destaque (30%), nos quais se privilegia o estudo da associação de constructos a partir de desenhos transversais, com o foco nos fatores de risco e de proteção ^14^ ^,^ ^36^ ^,^ ^37^ ^,^ ^38^ ^,^ ^39^ ^,^ ^42^ ^,^ ^43^ ^,^ ^60^ ^,^ ^62^ ^,^ ^65^ ^,^ ^66^ ^,^ ^68^ ^,^ ^70^ ^,^ ^71^ ^,^ ^73^ ^,^ ^74^ ^,^ ^75^ ^,^ ^76^ ^,^ ^77^ ^,^ ^78^ ^,^ ^82^ ^,^ ^84^ ^,^ ^91^ ^,^ ^100^ ^,^ ^109^ ^,^ ^110^ ^,^ ^112^ ^,^ ^113^ ^,^ ^114^. Outros ainda elencam estratégias e programas com forte evidência científica e testagem de eficácia ^40^ ^,^ ^54^ ^,^ ^56^ ^,^ ^115^ ^,^ ^116^. Um trabalho apresenta a validação de uma escala sobre dificuldade de regulação emocional ^45^. As intervenções estudadas são avaliadas no contexto escolar, comunitário ou clínico ^63^ ^,^ ^83^ ^,^ ^85^ ^,^ ^89^ ^,^ ^108^.

As ações se voltam à aplicação de estratégias/programas de prevenção de suicídio/autolesão com adolescentes com foco no fortalecimento das habilidades socioemocionais ^13^ ^,^ ^57^ ^,^ ^70^; na implementação de ações voltadas para adolescentes em risco ^61^; no auxílio e na capacitação de professores para a detecção do comportamento suicida/autolesivo ^57^ ^,^ ^91^; e na aplicação de intervenção baseada no tratamento, com ênfase na terapia comportamental dialética ^114^.

Há uma tendência de constatação dos efeitos positivos da aplicação de intervenções baseadas na inteligência emocional em adolescentes com histórico de comportamento suicida ou de autolesão ^83^ ^,^ ^85^ ^,^ ^89^ ^,^ ^108^, especialmente em contextos escolares e comunitários ^87^ ^,^ ^106^. Contudo, são poucos os que se baseiam em métodos que mostram evidências robustas de resultados ^11^ ^,^ ^86^. As estratégias abordadas se baseiam na revelação de emoções e/ou sentimentos, resolução de conflitos e busca de soluções alternativas diante das adversidades. Inclui a capacidade de interação social, que requer empatia, reciprocidade, cooperação e estratégias de negociação, além de bons relacionamentos ^44^ ^,^ ^53^ ^,^ ^57^ ^,^ ^69^ ^,^ ^80^. Munidos de tais habilidades, os adolescentes estariam mais preparados para enfrentar os desafios cotidianos, sociais e individuais, bem como os fatores de risco associados ao comportamento suicida à autolesão ^60^ ^,^ ^64^ ^,^ ^74^ ^,^ ^83^ ^,^ ^117^.

### O protagonismo da escola

É consenso o papel da escola como local privilegiado para trabalhar com as emoções como estratégia de prevenção do comportamento suicida e da autolesão ^52^. Com maior ou menor ênfase, é apresentada a necessidade de incluir a dimensão emocional como elemento indispensável ao desenvolvimento cognitivo, responsável pelo controle de sentimentos e emoções e indispensável na seleção de informações para guiar o pensamento e as ações no exercício social e cultural ^35^ ^,^ ^48^ ^,^ ^54^ ^,^ ^85^ ^,^ ^99^. O espaço escolar é estratégico para o desenvolvimento de ações de prevenção universal, ou seja, aquelas dirigidas a todos os públicos e voltadas para a aquisição de competências de aceitação e de tolerância emocional, promovendo uma atmosfera escolar de validação emocional ^35^ ^,^ ^46^ ^,^ ^57^ ^,^ ^76^ ^,^ ^87^ ^,^ ^99^. São também indicados programas de prevenção seletiva, em que o público-alvo é aquele que já apresenta sinais de comportamento suicida ou autolesão ^35^ ^,^ ^101^ ^,^ ^115^, ajudando os adolescentes a lidarem com os estados emocionais negativos resultantes de suas dificuldades interpessoais e a evitarem o seu impacto negativo na saúde mental ^46^ ^,^ ^76^ ^,^ ^87^ ^,^ ^99^ ^,^ ^106^.

Ações voltadas aos pais e professores são essenciais para o desenvolvimento de suas próprias habilidades socioemocionais e a dos adolescentes ^7^ ^,^ ^92^. A partir da perspectiva da psicoeducação, é indicada a necessidade de informá-los sobre os efeitos negativos de relações interpessoais focadas na ameaça, criticismo, subordinação e desvalorização.

Destacam-se os efeitos positivos do clima escolar, da importância do desenvolvimento de espaços de escuta e do fomento de mecanismos para que os estudantes desenvolvam habilidades socioemocionais ^51^ ^,^ ^53^. Em geral, as intervenções aplicadas na escola são desenvolvidas a partir de um currículo padrão, adaptado à realidade escolar e sociocultural. Currículos mais longos (não necessariamente em horas, mas ao longo do tempo) parecem se sustentar melhor, assim como aqueles que capacitam funcionários e professores da escola. Atuar no ambiente escolar reduz a violência, melhora o aprendizado, aumenta a permanência do adolescente na escola, facilita a relação entre pares e reduz o sofrimento - elementos importantes para o bem-estar e, consequentemente, para a prevenção do comportamento suicida.

Entre as ações destacadas no espaço escolar, está o treinamento da equipe de educação para que atue como guardiões, criando um ambiente escolar de apoio, reconhecendo fatores de risco e sinais de alerta de comportamento suicida, dando apoio a discentes angustiados e viabilizando ações colaborativas para obter suporte adicional para apoiar aqueles em sofrimento ^92^ ^,^ ^115^. Além disso, preconizam a promoção da saúde mental dos funcionários (treinamento e acesso a apoio) ^(2,52,92_^ e a educação sobre o uso saudável da internet e das mídias sociais ^51^. Também abordam o estímulo a um ambiente escolar seguro, como programas anti-*bullying* e iniciativas para aumentar a conexão social ^92^ ^,^ ^93^ ^,^ ^112^. Criar, fortalecer e divulgar contatos com serviços de suporte externo e prover uma política e protocolos claros para a equipe quando o risco de suicídio é identificado são outras ações importantes. Além disso, é fundamental apoiar o aluno a retornar à escola após uma tentativa de suicídio ^92^ ^,^ ^96^. Os pais precisam ser incluídos em todo processo para aumentar a conscientização sobre saúde mental ^57^ ^,^ ^82^. É destacada, ainda, a necessidade de discussão precoce sobre o comportamento suicida e o gerenciamento do estresse, com estímulo da regulação emocional e de ações antiestigma ^88^. Quanto mais precoce for a prevenção e a intervenção, melhor será o resultado. Outra recomendação é a criação de espaços nos quais crianças, adolescentes e pais/responsáveis possam procurar ajuda, aconselhamento, informação e ferramentas *online* ^74^.

### Aplicação das ações em serviços de saúde

As estratégias na saúde parecem decorrer da ação na educação, apresentando a reflexão do papel da intersetorialidade. A situação remete ao cenário no qual aqueles que “não deram certo a partir das ações da escola” chegam ao sistema de saúde já em situação agravada; ou seja, as ações de prevenção na saúde seriam seletivas ou indicadas, e as ações escolares seriam basicamente universais. É relevante incorporar o sistema de saúde na educação de base. Em relação à aplicação no Sistema Único de Saúde (SUS), as estratégias de cuidados não podem perder de vista os princípios da promoção e da atenção primária em saúde ^3^ ^,^ ^98^ ^,^ ^99^ ^,^ ^102^. Também focar na identificação de “sinais de alerta” para que os jovens possam ter ações alternativas de enfrentamento à sua disposição nos programas de saúde mental e em grupos de apoio. Essas iniciativas podem envolver as escolas, atuando no treinamento de “guardiões”, de colegas e adultos para reconhecer os sinais de alerta para o suicídio. Também podem trabalhar a cultura de uma escola em relação ao bem-estar psicológico e fazer triagem para identificar aqueles que podem estar em risco ^67^ ^,^ ^81^ ^,^ ^85^ ^,^ ^101^.

As principais estratégias para a atenção primária em saúde são: (i) promoção de saúde na comunidade, com garantia do pleno exercício do direito à saúde integral desde o acesso universal a serviços que promovam equidade e cobertura efetiva; (ii) garantia de cuidados integrais, integrados, adequados, de qualidade e sustentados ao longo do tempo; (iii) desenvolvimento de mecanismos de articulação intersetorial e participação de todos os atores da comunidade no planejamento e desenvolvimento de intervenções; e (iv) criação de metodologias participativas de planejamento e execução com o objetivo de identificar problemas prevalentes do território, mapear os atores e recursos presentes, abordar as condições de emergência que surgem, detectar e potencializar os fatores de proteção dos indivíduos em sua singularidade e das comunidades, e avaliar processos e resultados ^49^ ^,^ ^51^ ^,^ ^102^.

Há também debate sobre a necessidade de uma abordagem transversal a todos os níveis do sistema de saúde. Parece ser essencial haver um posicionamento estratégico das equipes no primeiro nível de atenção, implantando uma função de mediação e coordenação que efetivamente transforme a pirâmide do cuidado em rede, na qual as relações entre a equipe de saúde e outros serviços e instituições sejam baseadas em ações de cooperação ^54^ ^,^ ^77^ ^,^ ^96^ ^,^ ^102^. É crucial priorizar e implementar intervenções intra e interinstitucionais em acordo com a realidade de cada território e às histórias pessoais e comunitárias, as quais devem ser acomodadas para a restituição e o fortalecimento do vínculo social ^54^ ^,^ ^102^ ^,^ ^118^.

### Ações governamentais, não governamentais e o papel da mídia

Ações em nível governamental são cruciais para a prevenção do comportamento suicida e da autolesão pelo potencial de criar e implementar legislação nos níveis municipal, estadual e federal, a exemplo da restrição a meios letais. Ademais, é relevante a intervenção que inclui a participação de toda a sociedade e de instituições que trabalham com crianças e adolescentes, incluindo: realização de oficinas, fóruns, mesas redondas envolvendo professores, pais/responsáveis e crianças/adolescentes; articulação dos órgãos governamentais da três esferas (municipal, estadual e federal) com escolas, organizações juvenis, clubes, centros recreativos, organizações não governamentais (ONG) etc.; realização de pesquisas que elucidem temas estratégicos em nível local; identificação e capacitação de tutores/acompanhantes/líderes adolescentes, que possam ser referência em suas comunidades e promover processos de transferência de conhecimento e aprendizagem dos pares; divulgação de recursos culturais, recreativos e educativos para crianças e adolescentes; implementação de dispositivos de consultoria de saúde mental nas escolas; e campanhas de prevenção e conscientização na mídia e em espaços públicos ^77^ ^,^ ^102^ ^,^ ^104^ ^,^ ^116^.

A mídia foi destacada em número significativo de publicações como parceira no processo educativo de prevenção. Também é indicada como local estratégico no debate e promoção de ações sobre regras de comunicação na mídia sobre eventos suicidas ^2^ ^,^ ^77^ ^,^ ^95^. É dada ênfase ao seu papel na conscientização e redução do estigma de suicídio, no sentido de restringir informações sobre os meios e ambientes/locais propícios do ato suicida, sem dar visibilidade a qualquer descrição do método usado para se machucar de propósito ou se matar. Sugere-se que a mídia pode ajudar por meio da divulgação de informações sobre onde e como procurar ajuda e desenvolver diretrizes da cobertura responsável do suicídio, capacitando seus profissionais para relatos de casos. Desenvolver políticas para monitorar o conteúdo do usuário nas plataformas de mídia digital, criar páginas da *web* projetadas para ajudar os jovens a gerenciarem ou reduzirem a ideação suicida ou autolesão, e promover a interação social, permitindo o apoio de colegas, são outras ações citadas pela literatura ^2^ ^,^ ^3^ ^,^ ^51^ ^,^ ^93^ ^,^ ^99^ ^,^ ^102^ ^,^ ^119^.

## Discussão

No Brasil e no mundo, ainda é pouco conhecido o que pode ser feito para prevenir o comportamento suicida e a autolesão; apesar de diferentes estratégias serem descritas, poucas são claramente testadas e validadas. São muitas as dificuldades e impasses dos serviços de saúde, das escolas e de proteção social para atuarem quando se deparam com crianças e adolescentes que se ferem de propósito, pensam em se matar ou tentam cometer suicídio. A revisão de escopo realizada mostra que: (i) a maioria das iniciativas existentes é internacional; (ii) a maior parte do conhecimento produzido é voltado para o suicídio, poucos tratam exclusivamente da autolesão e outros agregam ambos, destacando pouco suas diferenças; (iii) as escolas são protagonistas no desenvolvimento de ações preventivas e a saúde precisa ampliar suas ações e atuar em rede colaborativa; e (iv) os principais temas desenvolvidos nas ações de prevenção no mundo voltam-se para a identificação precoce do comportamento suicida e da autolesão, promoção da vida socioemocional, limitação do acesso aos meios, apoio da mídia e foco na saúde mental.

A revisão de escopo revela que as ações voltadas às habilidades socioemocionais são estratégicas, pois auxiliam no processo de expressão e produção de emoções, atuando no significado da situação e na modulação da resposta emocional. Envolvem componentes fisiológicos, cognitivos, comportamentais e experienciais que variam em intensidade e avaliação subjetiva do indivíduo e usualmente podem ser provocados por situações interpessoais e fatos que merecem atenção porque afetam o bem-estar ^120^. Dessa forma, o adolescente modifica a maneira de se expor a determinadas situações, podendo avaliar melhor um problema, diminuir a tensão e flexibilizar a resposta cognitiva e emocional dos fatos ^42^ ^,^ ^118^ ^,^ ^120^.

Cabe destacar a importância de ações direcionadas aos pais e professores como fundamentais para a prevenção a partir das habilidades socioemocionais ^7^ ^,^ ^92^. Ademais, a colaboração intersetorial com atores governamentais e não governamentais é essencial para o cuidado precoce, o monitoramento e a avaliação dos casos ^2^ ^,^ ^3^. As áreas da saúde, da educação e da assistência social precisam atuar conjuntamente, uma vez que são estratégicas no cuidado diário de crianças e adolescentes com sinais precoces ou já instalados do comportamento suicida e da autolesão. Ações significativas têm sido propostas num debate pela busca de criação de ambientes de proteção a partir da redução do acesso a meios letais. No país, a Política Nacional de Prevenção da Automutilação e do Suicídio, por meio da *Lei nº 13.819/2019* ^121^, representa um marco legal importante e enfatiza a necessidade de estratégias de prevenção, mas não garante avanços na atenção em saúde mental.

Em geral, os programas de prevenção têm um tom de dar dicas e de como lidar com situações e poucos detalham a implementação de suas ações. Falham muito em apresentar resultados e critérios de avaliação. Dessa forma, é fortemente recomendado o registro das ações e resultados obtidos. Outra fragilidade diz respeito ao debate e aos achados primários sobre ações de prevenção específicas para grupos vulnerabilizados, como a população LGBTQIA+, pessoas desempregadas, migrantes, em privação de liberdade ou de cor de pele preta, sendo áreas nas quais o debate despontou de forma tímida no amplo acervo investigado.

A revisão apresentada tem como forte ponto positivo a grande amplitude da busca bibliográfica, com a inclusão da literatura de fontes estratégicas, o que dá um caráter inédito ao estudo, além da ênfase da prevenção a partir dos aspectos socioemocionais.

Por fim, em muitas partes do mundo e especialmente no Brasil, ainda não há orientações claras para a prevenção do comportamento suicida e da autolesão. O Estado precisa assumir seu protagonismo na orientação do cuidado e da proteção da população de crianças, adolescentes e suas famílias em sofrimento. O país ainda não avançou na elaboração de um plano nacional e de planos locais de prevenção, com orientações distintas para cada um desses comportamentos e operacionalização de uma abordagem prática e efetiva, enfatizando o papel do SUS, do cuidado em saúde mental e da articulação intersetorial para a promoção da saúde e qualidade de vida, assim como na prevenção do sofrimento e dos transtornos mentais.
